# Breakpoint Analysis of Transcriptional and Genomic Profiles Uncovers Novel Gene Fusions Spanning Multiple Human Cancer Types

**DOI:** 10.1371/journal.pgen.1003464

**Published:** 2013-04-25

**Authors:** Craig P. Giacomini, Steven Sun, Sushama Varma, A. Hunter Shain, Marilyn M. Giacomini, Jay Balagtas, Robert T. Sweeney, Everett Lai, Catherine A. Del Vecchio, Andrew D. Forster, Nicole Clarke, Kelli D. Montgomery, Shirley Zhu, Albert J. Wong, Matt van de Rijn, Robert B. West, Jonathan R. Pollack

**Affiliations:** 1 Department of Pathology, Stanford University School of Medicine, Stanford, California, United States of America; 2 Department of Medicine, University of California San Francisco, San Francisco, California, United States of America; 3 Department of Pediatrics, Stanford University School of Medicine, Stanford, California, United States of America; 4 Department of Neurosurgery, Stanford University School of Medicine, Stanford, California, United States of America; University of Washington, United States of America

## Abstract

Gene fusions, like *BCR/ABL1* in chronic myelogenous leukemia, have long been recognized in hematologic and mesenchymal malignancies. The recent finding of gene fusions in prostate and lung cancers has motivated the search for pathogenic gene fusions in other malignancies. Here, we developed a “breakpoint analysis” pipeline to discover candidate gene fusions by tell-tale transcript level or genomic DNA copy number transitions occurring within genes. Mining data from 974 diverse cancer samples, we identified 198 candidate fusions involving annotated cancer genes. From these, we validated and further characterized novel gene fusions involving *ROS1* tyrosine kinase in angiosarcoma (*CEP85L/ROS1*), *SLC1A2* glutamate transporter in colon cancer (*APIP/SLC1A2*), *RAF1* kinase in pancreatic cancer (*ATG7/RAF1*) and anaplastic astrocytoma (*BCL6/RAF1*), *EWSR1* in melanoma (*EWSR1/CREM*), *CDK6* kinase in T-cell acute lymphoblastic leukemia (*FAM133B/CDK6*), and *CLTC* in breast cancer (*CLTC/VMP1*). Notably, while these fusions involved known cancer genes, all occurred with novel fusion partners and in previously unreported cancer types. Moreover, several constituted druggable targets (including kinases), with therapeutic implications for their respective malignancies. Lastly, breakpoint analysis identified new cell line models for known rearrangements, including *EGFRvIII* and *FIP1L1/PDGFRA*. Taken together, we provide a robust approach for gene fusion discovery, and our results highlight a more widespread role of fusion genes in cancer pathogenesis.

## Author Summary

Gene fusions represent an important class of cancer genes, created by rearrangements of the genome that bring together two different genes. Because they are unique to cancer cells, gene fusions are ideal diagnostic markers and therapeutic targets. While gene fusions were once thought restricted mainly to blood cancers, recent discoveries suggest they are more widespread. Here, we have developed an approach for mining DNA microarray data to detect the tell-tale signatures of gene fusions, as “breakpoints” occurring within the encoding DNA or expressed transcripts. We apply this approach to a large collection of nearly 1,000 human cancer specimens. From this analysis, we discover and verify twelve new gene fusions occurring in diverse cancer types. We verify that some of these rearrangements recur in other samples of the same cancer type (supporting a causal role) and that the cancers show dependency on the fusion for cancer cell growth. Notably, some of these fusions (e.g. *CEP85L/ROS1* in angiosarcoma) represent the first for that cancer type and thus provide important new biological insight. Some are also good drug targets (including rearrangements of *ROS1*, *RAF1*, and *CDK6* kinases), with clear implications for therapy.

## Introduction

During cancer development and progression, chromosomal rearrangements frequently lead to the juxtaposition of two previously separate genes. The resulting gene fusions often play major roles in oncogenesis and generally fall into two categories. In the first, promoter or enhancer elements are juxtaposed to a proto-oncogene resulting in aberrant overexpression of an oncogenic protein (e.g. *IGH/MYC*). In the second category, the coding sequences of two genes are combined leading to the formation of a chimeric protein with new or altered activity (e.g. *BCR/ABL1*) [1]. Pathogenic gene fusions characterize many hematological and mesenchymal neoplasms [2], [3]. However, recent studies have demonstrated that epithelial malignancies can also harbor recurrent gene fusions, including ETS rearrangements in prostate cancer and *EML4/ALK* in non-small cell lung cancer [4]–[6]. Notably, gene fusions are used clinically for diagnosis and prognostication and can be important therapeutic targets, for example imatinib targeting *BCR/ABL1* and crizotinib targeting *EML4/ALK* [7], [8].

Gene fusions frequently represent markers for specific cancer subtypes. For example, chronic myelogenous leukemia (CML) is characterized by the Philadelphia chromosome and the resulting *BCR/ABL1* gene fusion, while acute promyelocytic leukemia (APL) is characterized by *RARA* rearrangement [9]–[12]. However, certain gene fusions occur across multiple cancer types (i.e. “multi-tumor” rearrangements). For example, *ETV6/NTRK3* has been described in secretory breast cancer, congenital fibrosarcoma, acute myeloid leukemia, and other malignancies [13]–[16]. Similarly, oncogenic rearrangements of the RAF kinases, *RAF1* and *BRAF*, have been found in various cancers including pilocytic astrocytoma, melanoma, gastric cancer, and prostate cancer [17], [18]. Such multi-tumor rearrangements suggest that cancers arising from distinct cell types and tissues might nonetheless represent related disease entities belonging to a common molecular grouping.

Advancements in genomic technologies have facilitated gene fusion discovery. Next-generation genomic and transcriptome sequencing have been used to discover novel gene rearrangements in prostate cancer, lung cancer, colon cancer, and melanoma [19]–[26]. Microarray-based approaches have been used to discover novel gene fusions in gastric cancer, prostate cancer, and leukemia [4], [27], [28]. Furthermore, major genome centers and consortiums, including The Cancer Genome Atlas Project (TCGA) and the Wellcome Trust Sanger Institute, have been using these genomic methodologies to profile large numbers of cancer specimens and have made these data publicly available [29]–[31]. In the modern era of cancer genomics, a major goal will be to mine these large datasets for the discovery of novel pathogenic alterations that drive oncogenesis.

We hypothesized that novel multi-tumor rearrangements exist across various cancers and should be discoverable in large genomic datasets. Here we describe the development of a pipeline for the detection of these alterations based on the identification of tell-tale rearrangement “breakpoints” in transcriptome and genomic data. We apply this method to both publicly-available microarray datasets as well as data generated in our laboratory. As a proof of concept, we successfully rediscovered several known gene fusions. More significantly, we nominate and subsequently validate several novel gene fusions spanning multiple human cancer types.

## Results

### Microarray datasets

For our breakpoint analysis (detailed below), we mined transcriptome data from 92 exon microarray experiments, together representing 12 different cancer types (Figure S1). Our laboratory generated 16 of these profiles, which included several specimens with known rearrangements to optimize our methodology, as well as various cancer types where gene fusions had yet to be discovered. The remaining data were obtained from published studies [32], [33]. In particular, we focused on datasets of established cancer cell lines, so that we could readily obtain the samples for validation and follow-up experiments. Separately, we mined genomic profiles from 882 high-density array-based comparative genomic hybridization (aCGH) experiments (Figure S1). Of these samples, 812 were generated from the Wellcome Trust Sanger Institute's Cancer Genome Project, which included cancer cell lines from 29 distinct tissue sites [31]. The remaining profiles were generated in our laboratory [34] and comprised 70 pancreatic cancer cell lines and early-passage xenografts.

### Breakpoint analysis

**Figure 1:**
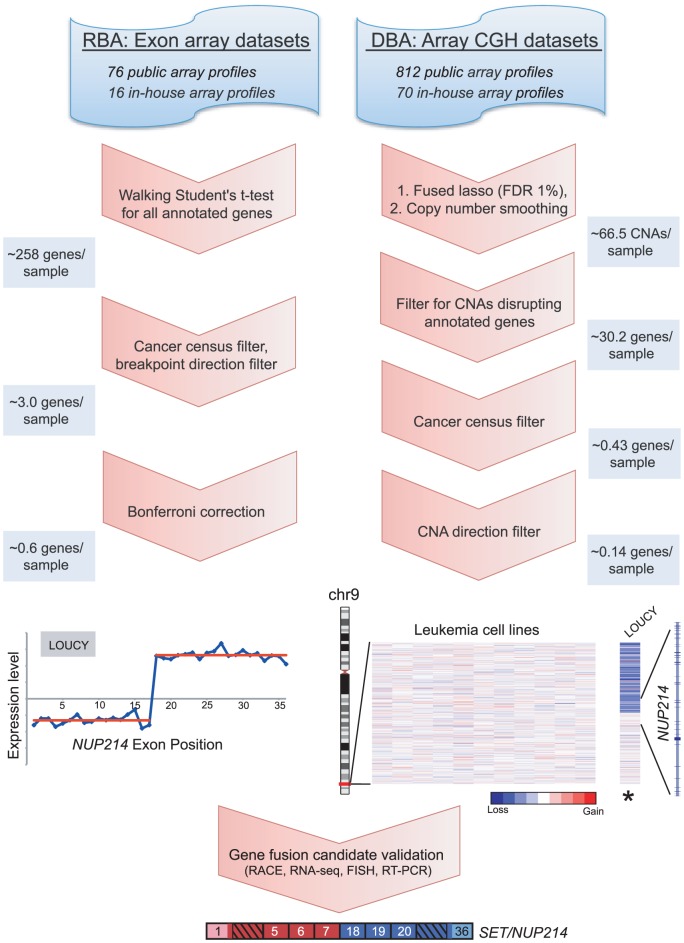
Breakpoint analysis for discovering novel cancer gene rearrangements. Schematic depiction of the approach and workflow, demonstrated by example of the rediscovery of a known gene fusion, *SET/NUP214*, in the T-ALL cell line LOUCY. Various publicly-available and in-house exon microarray and high-density CGH/SNP array experiments were analyzed. RNA breakpoint analysis (RBA) identifies significant transitions in exon expression level, which may reflect elevated expression of exons distal (3′ partner) or proximal (5′ partner) to a gene fusion junction. To identify such transitions a “walking” Student's t-test was applied, comparing expression levels of proximal and distal exons. Candidate rearrangements were subsequently filtered for those disrupting genes of the Cancer Gene Census, with directional orientation (i.e. being the 5′ or 3′ partner) consistent with known rearrangements of that gene. RBA candidates were further filtered using a Bonferroni correction to adjust for multiple t-tests. DNA breakpoint analysis (DBA) screens for intragenic DNA copy number transitions, which may reflect unbalanced chromosomal rearrangements leading to the formation of gene fusions. The fused lasso method (FDR 1%) followed by a copy number smoothing algorithm was applied to identify CNAs. Copy number transitions were filtered for those disrupting any annotated gene and then further filtered for those disrupting genes of the Cancer Gene Census. We included only candidate breakpoints where the directional orientation of the copy number transition was consistent with known rearrangements of that gene. Several candidates were then validated using molecular and cytogenetic approaches. The average numbers of candidate rearrangements per cancer sample are depicted along the left and right panels at various stages of the workflow.

To nominate candidate gene fusions from transcriptome data (using exon microarrays), we developed an approach which we termed RNA breakpoint analysis (RBA) (Figure 1 and Figure S2). Other groups have proposed similar methods, although in limited application to detect known fusions [35]–[37], or very recently with some success in discovering novel fusions [38], [39]. Our strategy was to identify transcript “breakpoints”, i.e. significant transitions in expression level between proximal and distal exons. These transitions might reflect elevated expression of the exons proximal (for 5′ fusion partners) or distal (for 3′ fusion partners) to a gene fusion junction. To identify such transitions, we implemented a “walking” Student's t-test, comparing expression levels of proximal and distal exons (testing all possible exonic breakpoints), for all assayed transcripts (Figure S2A, S2B). Because such transitions might be present due to reasons other than rearrangement (e.g. alternative splicing), we applied additional filters to enrich for true positives (see Materials and Methods), including applying a stringent Bonferroni correction to adjust for multiple gene testing. We also limited our analysis to candidate breakpoints that disrupted genes known to be rearranged in human cancer, as defined by the Cancer Gene Census [40]. Though we might miss some novel genes, we reasoned, as have others [4], that as a starting point this gene set would be enriched for true positives, and for novel “multi-tumor” gene fusions that might span multiple cancer types.

To discover gene fusions from genomic data (using high-density CGH/SNP arrays), we employed a similar method called DNA breakpoint analysis (DBA), based on identifying intragenic breakpoints as transitions in DNA copy number occurring within genes (Figure 1 and Figure S3A). These intragenic genomic breakpoints might reflect unbalanced chromosomal rearrangements that result in the creation of a gene fusion. Other groups have recently reported similar approaches, though in limited datasets, either to rediscover known gene fusions or to discover novel rearrangements [27], [28], [41]. To identify genomic breakpoints, we first segmented the copy number data to identify statistically-significant copy number alterations (CNAs), using the fused lasso method (false discovery rate; FDR 1%) [42]. Because this approach tended to overcall copy number transitions, we also devised an algorithm to better define the boundaries of statistically-significant CNAs, which we termed “copy number smoothing” (see Materials and Methods). We then screened for copy number changes disrupting those genes of the Cancer Gene Census.

Altogether, RBA identified 54 different transcript breakpoints across the 92 cancer samples analyzed (Figure S2C and Table S1). Many of these breakpoints corresponded to known gene fusions, including *BCR/ABL1* in CML, *FIP1L1/PDGFRA* in eosinophilic leukemia, and *NPM1/ALK* in anaplastic large cell lymphoma (ALCL) (Figure S4). In most cases of known gene fusions, we found that RBA was better suited to detect the 3′ fusion partner. This likely reflects that for 5′ partners, comparable expression of the remaining wildtype allele might mask an expression-level breakpoint, whereas for 3′ partners the corresponding wildtype allele is more likely to be expressed at low or negligible levels (from its endogenous promoter).

Altogether, DBA identified 144 different intragenic DNA copy number breakpoints across the 882 cancer samples analyzed (Figure S3B and Table S2). Many of these candidates also corresponded to known gene fusions, including *EWSR1/FLI1* in Ewing sarcoma and *ABL1* rearrangements in several leukemia samples (Figure S5). When possible, RBA and DBA results were integrated. In particular, four candidates were supported by both approaches, with three corresponding to known gene fusions (Table S1 and Table S2). However, opportunities for integrating RBA and DBA were few because of the limited overlap of samples profiled at both the transcriptional and genomic level.

In all, we prioritized two candidate gene fusions nominated by RBA and 12 candidate rearrangements nominated by DBA for further characterization. We used various criteria to select these candidates, and our rationale is presented in more detail in Table S1 and Table S2. Briefly, we prioritized RBA candidates by focusing on the most statistically-significant novel rearrangements. For DBA, we prioritized novel rearrangements associated with *focal* copy number alterations, because we noted in the datasets that many known gene fusions occurred in the context of focal genomic gains or losses. We also used gene-expression profiling data when available to prioritize DBA candidates that were highly expressed in the respective sample. In addition, for both RBA and DBA, we prioritized breakpoints aligning to exon positions previously demonstrated to be rearranged in other malignancies. In total, we were able to define and PCR-validate rearrangements in 12 of the 14 (86%) candidates tested (Table 1, Table S1, and Table S2).

**Table 1:** Validated gene fusions and rearrangements.

Gene fusion^a^	Sample	Type	Tissue type	Discovery method	No. supporting reads
*ABL1/CBFB* b	A172	Cell line	GBM	DBA	30
*APIP/SLC1A2*	SNU-C1	Cell line	Colon cancer	DBA	57
*ATG7/RAF1*	PL5	Cell line	Pancreatic cancer	DBA	14
*BCL6/RAF1*	D-538MG	Cell line	Anaplastic astrocytoma	DBA	39
*CEP85L/ROS1*	AS1	Tumor	Angiosarcoma	RBA	NA
*CLTC/VMP1*	BT549	Cell line	Breast cancer	DBA	16
*CLTC/VMP1*	HCC1954	Cell line	Breast cancer	DBA	95
*EGFR/PPARGC1A* b	A431	Cell line	Skin squamous cell carcinoma	DBA	46
*EGFRvIII*	DKMG	Cell line	GBM	DBA	16
*EWSR1/CREM*	CHL-1	Cell line	Melanoma	DBA	120
*FAM133B/CDK6*	J-RT3-3T-5	Cell line	T-ALL	DBA	30
*FIP1L1/PDGFRA*	SUPT13	Cell line	T-ALL	RBA	13

a Gene fusions initially nominated by breakpoint analysis and subsequently validated by paired-end RNA-seq (or 5′ RACE for *CEP85L/ROS1*) and RT-PCR.

b *ABL1* and *EGFR* locus rearrangements were previously reported in the respective cell lines [96]–[98]; however associated fusion transcripts were not identified.

### Novel *ROS1* rearrangements in angiosarcoma and epithelioid hemangioendothelioma

**Figure 2:**
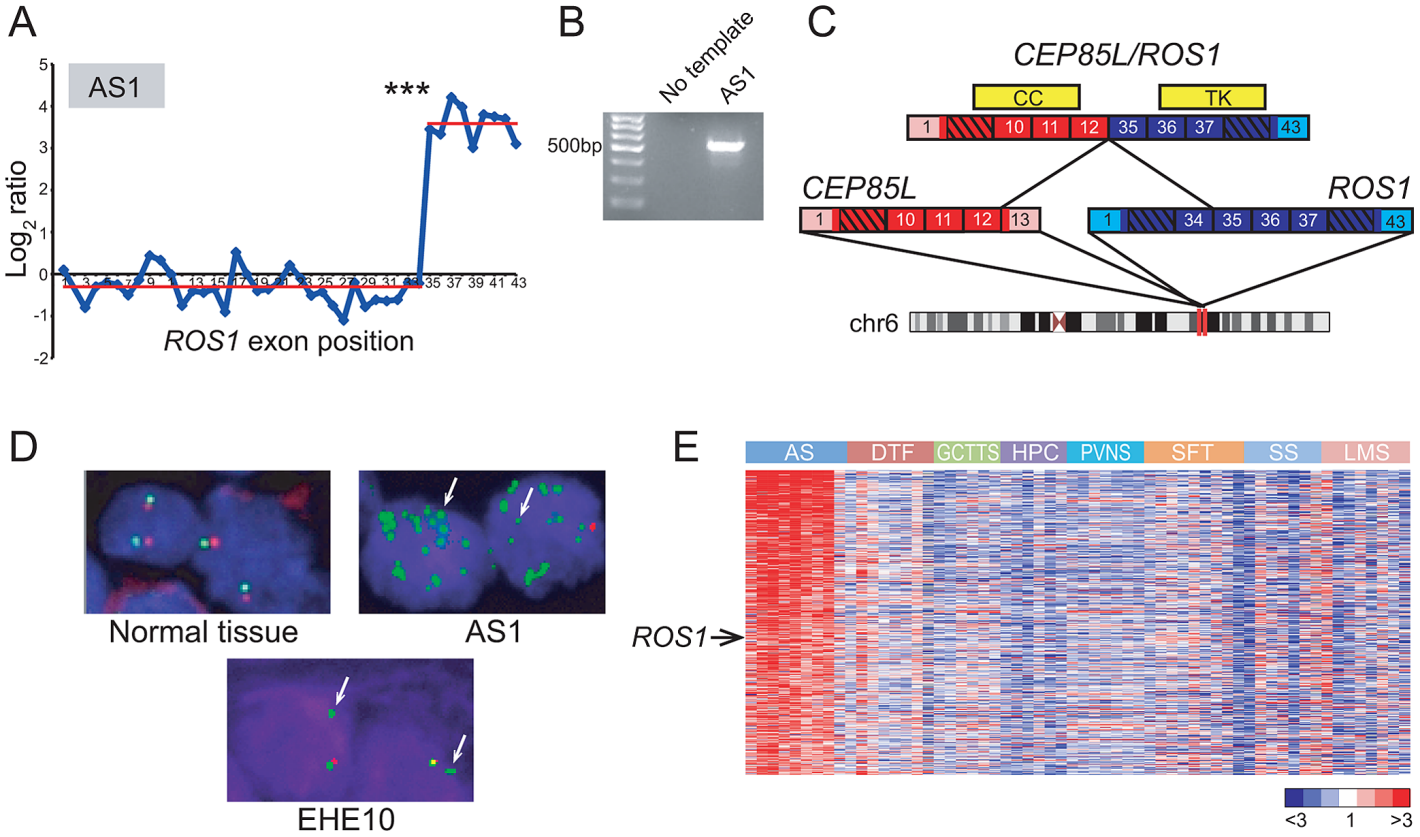
Discovery and characterization of *CEP85L/ROS1* in angiosarcoma. (*A*) RBA of angiosarcoma specimen AS1 reveals an expression breakpoint between exons 34 and 35 of *ROS1*, suggesting rearrangement. (*B*) Experimental validation of *CEP85L/ROS1* in AS1 by RT-PCR, using primers flanking the gene fusion junction. (*C*) Predicted structure of *CEP85L/ROS1*. *CEP85L* and *ROS1* are oriented in the same direction and located ∼1 MB apart within cytoband 6q22. The gene fusion preserves a coiled-coil (CC) domain from *CEP85L* and the tyrosine kinase (TK) domain of *ROS1*. Exons are numbered, with untranslated regions depicted in corresponding lighter shades. (*D*) Break-apart FISH demonstrates rearrangement of *ROS1* in angiosarcoma and epithelioid hemangioendothelioma. Co-localizing red and green signals are indicative of normal chr 6 (*left* panel). AS1 exhibits loss of red signal with multiple green signals indicative of amplification of rearranged *ROS1*. An epithelioid hemangioendothelioma specimen (EHE10) also exhibits loss of red signal, indicative of unbalanced rearrangement of *ROS1*. (*E*) Increased *ROS1* expression in angiosarcoma compared to other sarcoma subtypes. Heatmap depicts genes selectively overexpressed in angiosarcoma, identified by supervised analysis. Genes are ordered by rank value of their t-statistic scores. Mean-centered gene expression ratios are depicted by a log_2_ pseudocolor scale (ratio-fold change indicated). AS: angiosarcoma, DTF: desmoid-type fibromatosis, GCTTS: giant cell tumor-tendon sheath, HPC: hemangiopericytoma, PVNS: pigmented villonodular synovitis, SFT: solitary fibrous tumor, SS: synovial sarcoma, LMS: leiomyosarcoma. *** *P = 4.26×10^−28^* (Student's t-test).

Rare oncogenic gene fusions involving the *ROS1* receptor tyrosine kinase (RTK), a poorly characterized RTK with unknown ligand [43], have been described in glioblastoma, non-small cell lung cancer, and cholangiocarcinoma [44]–46. DBA identified a genomic breakpoint disrupting *ROS1* in U-118MG cells, corresponding to the known *GOPC/ROS1* (also called *FIG/ROS1*) gene fusion in this glioblastoma cell line [45] (Figure S5C). In addition, RBA nominated 6 other candidate *ROS1* rearrangements, in breast cancer (BT-549, HS578t), glioblastoma (SF-295, U251), lung cancer (HOP-62), and angiosarcoma (AS1). However, only the primary angiosarcoma specimen, AS1, exhibited a prominent and highly significant (*P*<10^−27^) expression transition (Figure 2A), with the predicted breakpoint corresponding to known rearrangements of *ROS1* in other malignancies. Thus, we chose to further investigate *ROS1* in this specimen.

While several sarcoma subtypes (e.g. Ewing sarcoma) harbor pathognomonic gene fusions, no such alterations have been discovered to date in angiosarcoma, a rare but aggressive endothelial neoplasm [47], [48]. By 5′ rapid amplification of cDNA ends (5′ RACE), we uncovered a novel *CEP85L/ROS1* rearrangement in AS1 (Figure 2B, 2C). *CEP85L* and *ROS1* are located approximately 1 megabase (MB) apart within cytoband 6q22, and are oriented in the same direction. The gene fusion is in frame, and preserves the tyrosine kinase domain of *ROS1*, but removes its transmembrane and extracellular domains (Figure 2C). *CEP85L* was recently discovered to be the 5′ partner of a rearrangement involving *PDGFRB* in a patient with precursor T-ALL and an associated myeloproliferative neoplasm [49]. The breakpoint of *CEP85L/PDGFRB* includes the first 11 exons of *CEP85L* whereas *CEP85L/ROS1* includes the first 12 exons. While little is known about the function of *CEP85L* (centrosomal protein 85 kDa-like), structural analysis [50] predicts the presence of a coiled-coil domain that is retained in these gene fusions (Figure 2C). Rearrangements of RTKs often involve 5′ (N-terminal) partnered coiled-coil domains, which, presumptively by mediating dimerization, are necessary for the transforming properties of these fusions [5], [51], [52].

To further investigate the underlying genomic rearrangement in the AS1 angiosarcoma specimen, we performed a “break-apart” fluorescence *in situ* hybridization (FISH) assay, using two FISH probes (with different fluors) flanking *ROS1*. FISH analysis confirmed genomic rearrangement with amplification of *ROS1* (Figure 2D). To determine whether *ROS1* rearrangements recurred in angiosarcomas or other sarcoma subtypes, we performed the break-apart FISH assay on two tissue microarrays (TMA) containing 280 specimens representing 36 diverse sarcoma and soft tissue tumor diagnoses (Table S3). An advantage of FISH (e.g. as compared to RT-PCR) is that it does not require knowing the identity of the 5′ fusion partner, which may differ among tumor specimens. Of 33 evaluable angiosarcoma and 20 epithelioid hemangioendothelioma (EHE; a related diagnosis) cases, one EHE case (EHE10) exhibited rearrangement at the *ROS1* locus (Figure 2D). Thus, in all we observed *ROS1* rearrangement in 1 of 34 (∼3%) angiosarcomas and 1 of 20 (5%) EHE cases. No additional *ROS1* rearrangements were identified in other sarcoma and soft tissue tumor subtypes.

Although *ROS1* rearrangements appeared to be relatively uncommon, we hypothesized that ROS1 might nonetheless play a more general role in angiosarcoma pathogenesis, even in cases without rearrangement. To explore this hypothesis, we analyzed a microarray dataset of gene-expression profiles from various sarcoma subtypes including angiosarcoma [53]–[55]. By supervised analysis, we identified 455 genes (FDR<5%) with elevated expression in angiosarcoma relative to other sarcoma subtypes. In addition to including various vascular endothelial markers (*ECSCR*, *TIE1*, *CD34*, *CDH5*, *ESAM*), the angiosarcoma gene signature also included *ROS1* (Figure 2E), supporting a possible broader role of ROS1 in the pathogenesis of this disease.

### Discovery of *APIP/SLC1A2* in colon cancer

Recently, Tao *et al.* reported that a small subset of gastric cancers harbors the novel gene fusion *CD44/SLC1A2* [27]. This fusion is formed through a chromosomal inversion that juxtaposes most of the coding region of the glutamate transporter gene *SLC1A2* to the strong transcriptional promoter of its neighboring gene *CD44*. The rearrangement results in overexpression of an N-terminally truncated SLC1A2 protein, which increases intracellular glutamate levels and stimulates oncogenic growth.

**Figure 3:**
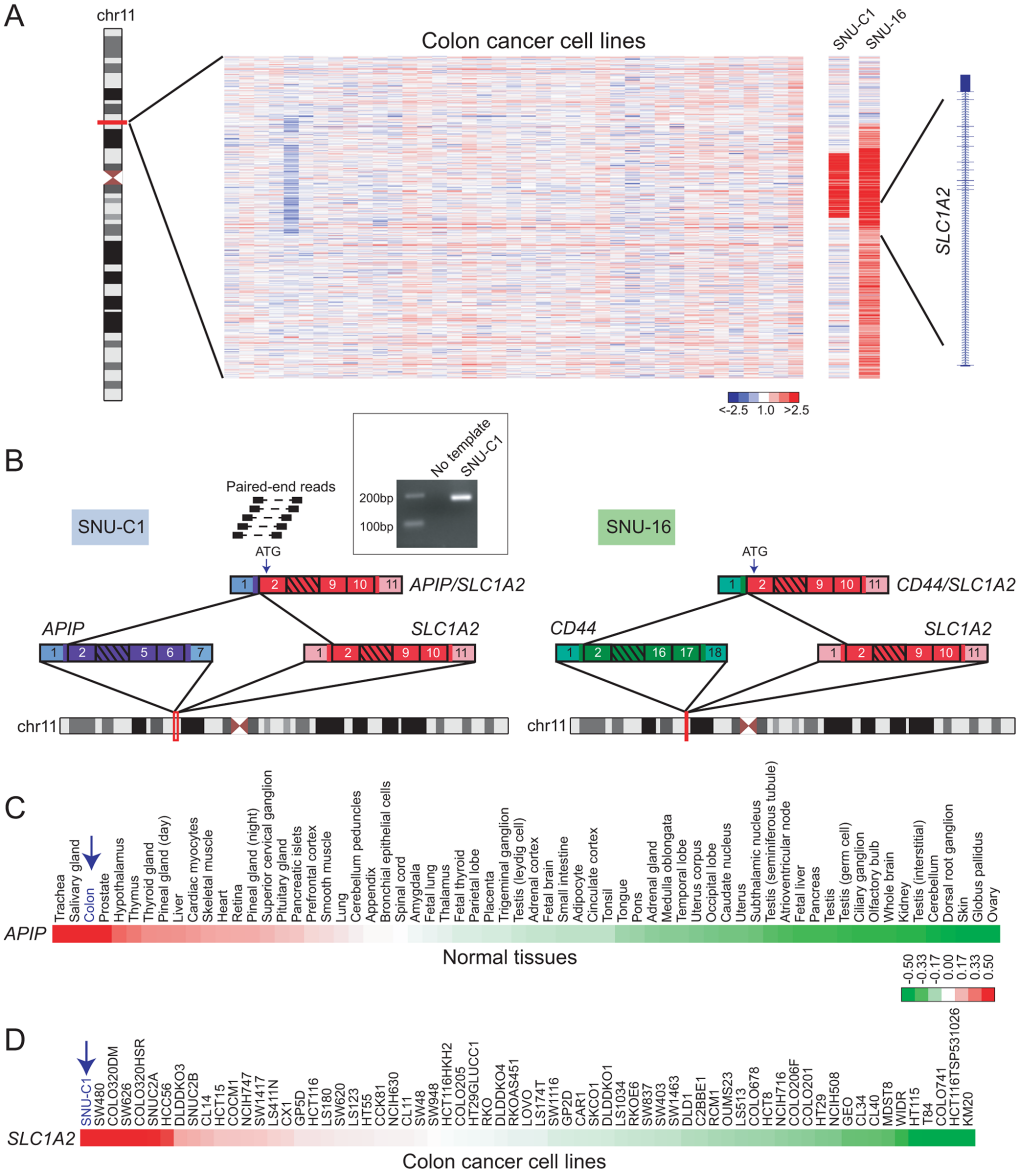
Discovery of *APIP/SLC1A2* in colon cancer. (*A*) Array CGH heatmap displaying genomic breakpoints disrupting *SLC1A2* in the SNU-C1 colon cancer cell line and the SNU-16 gastric cancer cell line. SNU-16 is known to harbor *CD44/SLC1A2* and its array CGH profile is depicted for comparison. Unsmoothed log_2_ ratios are displayed. (*B*) Paired-end RNA seq uncovers *APIP/SLC1A2* in SNU-C1. A subset of paired-end reads mapping to *APIP/SLC1A2* as well as the gene fusion structure are displayed (left panel). The structure of the known gastric cancer gene fusion *CD44/SLC1A2* is depicted for comparison (right panel). An internal start codon within exon 2 of *SLC1A2* is predicted to initiate translation in both rearrangements. *Inset*: experimental validation of *APIP/SLC1A2* by RT-PCR with primers flanking the gene fusion junction. (*C*, *D*) Gene expression profiling depicts high-level expression of *APIP* in normal colon (*C*) and overexpression of *SLC1A2* in SNU-C1 (*D*). Mean-centered gene expression ratios are depicted by a log_2_ pseudocolor scale and ranked in descending order from left to right.

Our DBA results suggested that *SLC1A2* rearrangements occur in cancer types other than gastric carcinomas. In addition to detecting a known *SLC1A2* rearrangement in the gastric cancer cell line SNU-16, DBA identified breakpoints disrupting *SLC1A2* in the colon cancer cell line SNU-C1 and in a pancreatic cancer xenograft (247) (Figure 3A and Table S2). All of these breakpoints occur within intron 1 of *SLC1A2*, the same position found to be disrupted in several gastric cancers [27]. We chose to further characterize the putative rearrangement in SNU-C1.

By paired-end RNA sequencing (RNA-seq; see Materials and Methods) of SNU-C1 cells, we uncovered a novel colon cancer gene fusion, *APIP/SLC1A2* (Figure 3B). The structure of this rearrangement is nearly identical to that of *CD44/SLC1A2* and is predicted to encode the same truncated transporter protein. In particular, as is the case for *CD44/SLC1A2*, translation is predicted to occur from an internal start codon within exon 2 of *SLC1A2* (Figure 3B).

Analogous to *TMPRSS2/ERG* in prostate cancer, the *SLC1A2* fusion in gastric cancer is thought to be driven by strong expression of its 5′ partner, *CD44* [27]. We therefore reasoned that for *APIP/SLC1A2*, the 5′ partner *APIP* (APAF1 interacting protein) ought to exhibit strong expression in colon. Indeed, analysis of publicly-available microarray data [56], [57] revealed high-level expression of *APIP* in colon compared to other tissues (Figure 3C). Furthermore, analysis of a publicly-available colorectal cancer gene-expression dataset [58] demonstrated *SLC1A2* to be expressed at higher levels in SNU-C1 compared to all other cell lines interrogated (Figure 3D). Attempts to characterize the oncogenic contribution of *APIP/SLC1A2* by RNA interference (RNAi)-mediated knockdown were met with technical difficulties in efficiently transfecting the suspension line SNU-C1 (data not shown). Further studies are needed to fully characterize the role of this alteration in colon carcinogenesis.

### Novel RAF kinase rearrangements in pancreatic cancer and anaplastic astrocytoma

**Figure 4:**
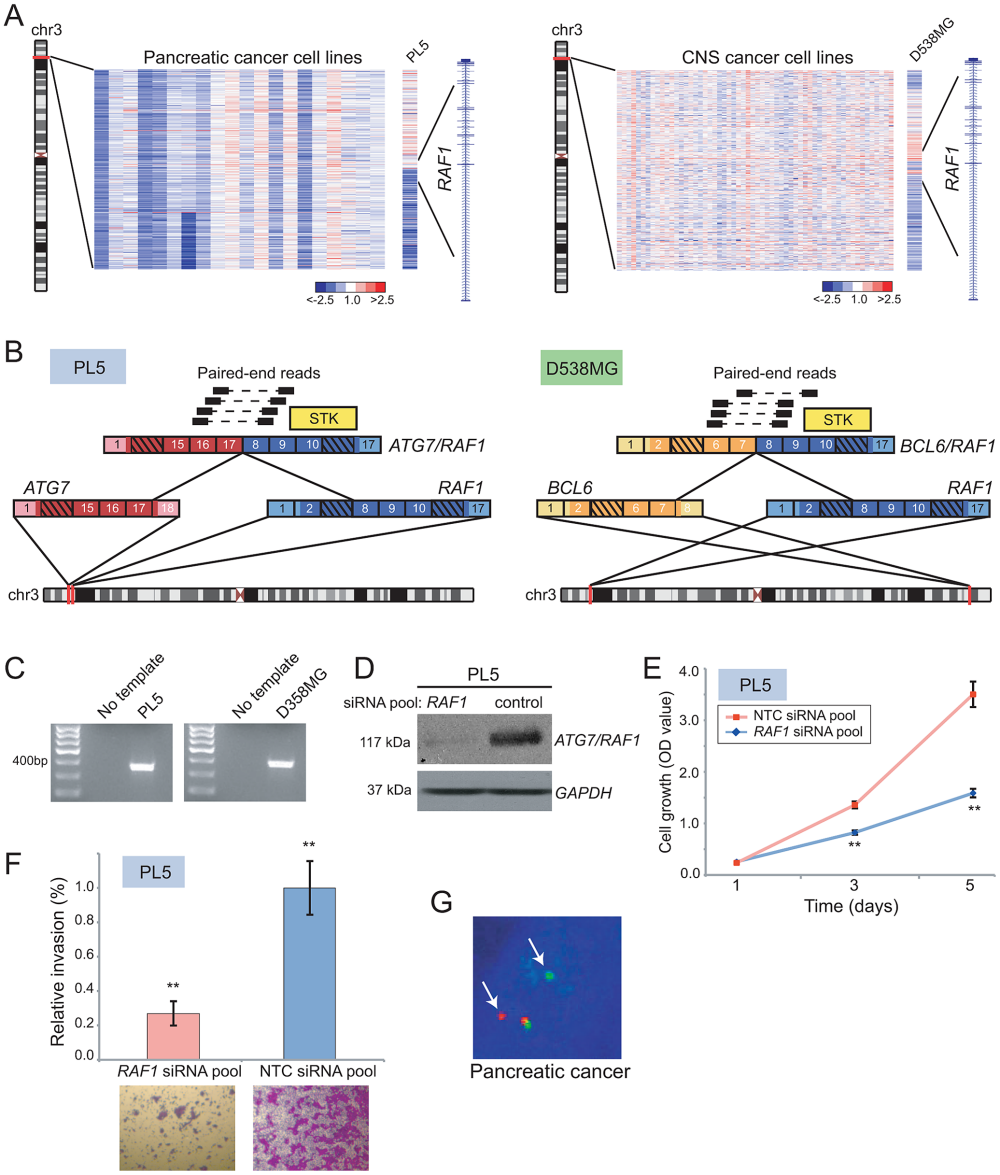
Identification and characterization of novel *RAF1* gene fusions in pancreatic cancer and anaplastic astrocytoma. (*A*) Array CGH heatmaps displaying intragenic *RAF1* genomic breakpoints identified in the PL5 pancreatic cancer cell line (*left panel*) and the D-538MG anaplastic astrocytoma cell line (*right panel*). Unsmoothed log_2_ ratios are displayed. (*B*) Identification of *ATG7/RAF1* (left) and *BCL6/RAF1* (right) in PL5 and D-538MG cells, respectively, by paired-end RNA-seq. A subset of the paired-end reads supporting each gene fusion is displayed. Both gene fusions are in-frame and the *RAF1* serine threonine kinase domain (STK) is retained in both fusions. (*C*) Experimental validation of gene fusions by RT-PCR, using primers flanking the respective gene fusion junction. (*D*) Western blotting verifies knockdown of ATG7/RAF1 in PL5 following transfection of a *RAF1*-targeting siRNA pool. ATG7/RAF1 protein levels were monitored using an anti-*RAF1* antibody, with anti-*GAPDH* providing a loading control. (*E*) Decreased cell proliferation and (*F*) invasion rates of PL5 following transfection of a *RAF1*-targeting siRNA pool, compared to transfection of a non-targeting control (NTC) siRNA pool. ** *P*<0.01 (two-sided Student's t-test). (*G*) Break-apart FISH demonstrates rearrangement of *BRAF* in a pancreatic cancer case from the TMA, as evidenced by physical separation of the red and green probes (arrows) flanking *BRAF* (single interphase nucleus shown).

Recurrent rearrangements of the RAF kinases, *RAF1* and *BRAF*, were recently reported in a small fraction of prostate cancers, gastric cancers, and melanomas [17]. Here, DBA identified candidate rearrangements of *RAF1* in lung cancer (DMS-153), pancreatic cancer (PL5), anaplastic astrocytoma (D538-MG), and osteosarcoma (CAL-72) (Figure 4A and Table S2), and candidate rearrangements of *BRAF* in gastric cancer (NCI-N87), breast cancer (HCC38), and glioblastoma (D397-MG) (Table S2). We further evaluated two of these candidates by paired-end RNA-seq, from which we identified novel gene fusions, *ATG7/RAF1* in pancreatic cancer and *BCL6/RAF1* in anaplastic astrocytoma (Figure 4B, 4C). Both of these fusions retained exons 8–17 of *RAF1*, and the encoded fusions were predicted to be in frame. In addition, both rearrangements preserved the RAF1 serine/threonine kinase domain but removed an N-terminal autoinhibitory Ras Binding Domain (RBD) (Figure 4B), consistent with the structural organization of known RAF kinase gene fusions [17], [18].

We further characterized the oncogenic relevance of *ATG7/RAF1* in pancreatic cancer, using RNAi to knockdown its expression. Transfection of PL5 cells with short interfering RNAs (siRNAs) targeting the 3′ end of *RAF1* (i.e. the portion retained in the fusion) led to reduced expression of the RAF1 fusion (Figure 4D). This resulted in significantly decreased cell proliferation and invasiveness (by Boyden chamber assay), compared to PL5 cells transfected with a non-targeting control siRNA (Figure 4E, 4F).

More than 90% of pancreatic cancers harbor activating mutations of *KRAS*, and a subset also exhibits *KRAS* amplification [59]. Comparatively little is known of the pathobiology of the pancreatic cancer subset that is wildtype for *KRAS*. Since RAF kinases mediate KRAS signaling through the MAPK cascade, we reasoned that *ATG7/RAF1* might substitute for *KRAS* mutation in PL5. Supporting this possibility, PL5 exhibited neither amplification (by aCGH profile; data not shown) nor activating mutation (by Sanger sequencing) of *KRAS*.

To determine whether RAF kinase rearrangements are recurrent events in pancreatic cancer, we performed break-apart FISH assays for both *BRAF* and *RAF1*, on TMAs containing 104 evaluable pancreatic cancer cases. We identified *BRAF* rearrangement in one of the 104 samples (∼1%) (Figure 4G) but no additional *RAF1* rearrangements. Taken together, our findings are consistent with RAF kinase fusions occurring in a small subset of pancreatic cancers, where they possibly substitute for *KRAS* mutations.

### Discovery and characterization of *EWSR1/CREM* in melanoma

**Figure 5:**
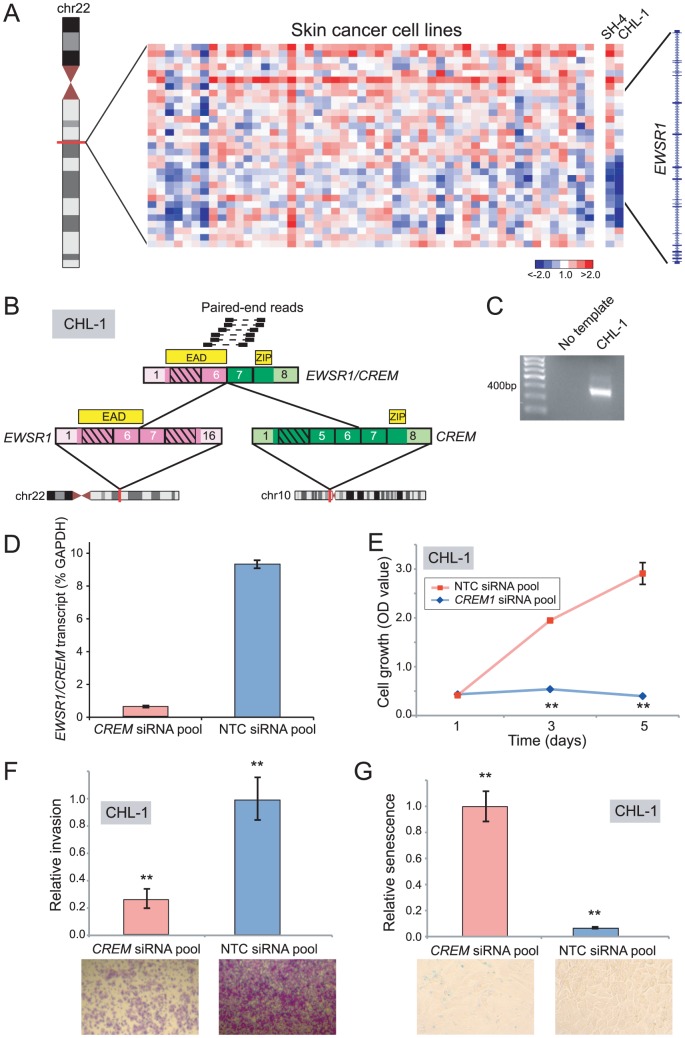
Discovery and characterization of *EWSR1/CREM* in melanoma. (*A*) Array CGH heatmap displaying intragenic *EWSR1* breakpoints identified in the SH-4 and CHL-1 melanoma cell lines. (*B*) Paired-end RNA-seq identification of *EWSR1/CREM* in CHL-1. Paired-end reads supporting the rearrangement are depicted along with the predicted gene fusion structure. CREM contributes a basic leucine zipper motif (ZIP), while EWSR1 contributes the EWS Activation Domain (EAD). (*C*) RT-PCR verification of *EWSR1/CREM* in CHL-1. (*D*) Quantitative RT-PCR using primers flanking the gene fusion junction verifies *EWSR1/CREM* knockdown following transfection of an siRNA pool targeting the 3′ end of *CREM*. (*E*, *F*, *G*) Transfection of CHL-1 with *CREM*-targeting siRNA pool results in (*E*) decreased cell proliferation, (*F*) decreased invasion, and (*G*) a higher fraction of senescent cells, compared to non-targeting control (NTC). **P<0.01 (two-sided Student's t-test).

Rearrangements of the RNA binding protein, *EWSR1*, characterize various malignancies including Ewing sarcoma (*EWSR1/ETS*), desmoplastic small round cell tumor (*EWSR1/WT1*), and some acute lymphoblastic leukemias (*EWSR1/ZNF384*) [60]–[62]. By DBA, we identified intragenic breakpoints disrupting *EWSR1* in Ewing sarcoma (ES6, EW12, EW22), neuroblastoma (GOTO, NBsusSR), and melanoma (CHL-1, SH4) (Figure 5A and Table S2). As *EWSR1* gene fusions had not previously been described in cutaneous melanoma, we prioritized CHL-1 and SH4 for further evaluation.

By paired-end RNA-seq, we uncovered a novel rearrangement, *EWSR1/CREM*, in CHL-1 (Figure 5B, 5C), but were unable to identify an *EWSR1* fusion in SH4. CREM is a basic leucine zipper transcription factor and downstream mediator of the cAMP signal transduction cascade [63]–[65]. The structure of *EWSR1/CREM* is typical of oncogenic *EWSR1* rearrangements, with a putative transcriptional transactivating domain from *EWSR1* fused in-frame to the basic leucine zipper DNA binding domain of *CREM* (Figure 5B).

To explore an oncogenic contribution of *EWSR1/CREM* in melanoma, we again used RNAi to knockdown expression of the fusion. Transfection of CHL-1 cells with siRNAs targeting the 3′ end of *CREM* (the portion retained in the fusion) led to reduced transcript levels of the *EWSR1/CREM* fusion (Figure 5D), and to significantly decreased cell proliferation and invasion (compared to non-targeting control siRNAs) (Figure 5E, 5F). Notably, CHL-1 cells transfected with *CREM*-targeting siRNAs also appeared flattened and enlarged, morphological changes suggestive of senescence. To substantiate this observation, we stained for senescence-associated β-galactosidase and observed significantly increased numbers of senescent cells (Figure 5G).

### Identification of *FAM133B/CDK6* in T-ALL

**Figure 6:**
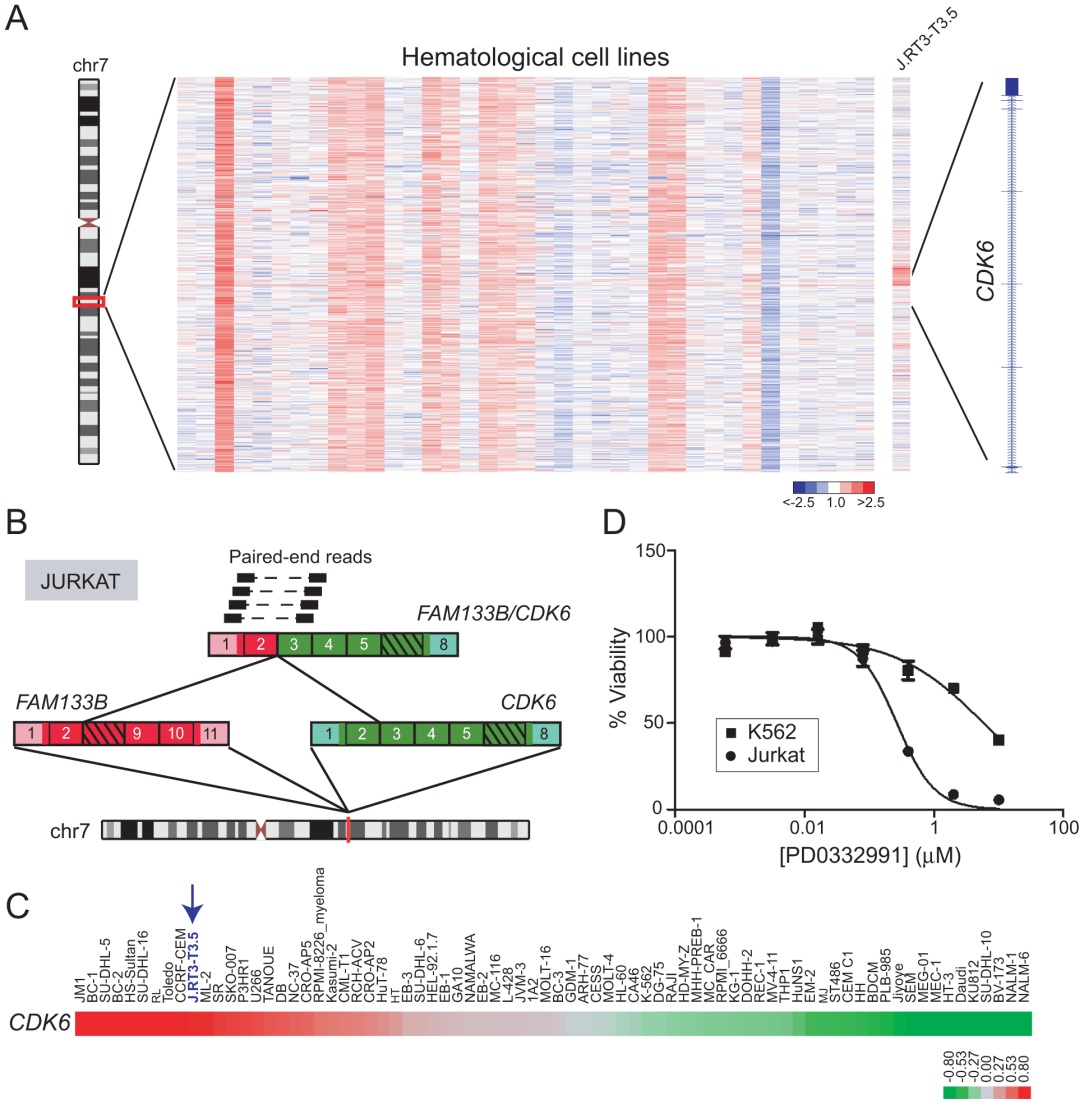
Identification and characterization of *FAM133B/CDK6* in J.RT3-T3.5. (*A*) Heatmap depicting rearrangement of *CDK6* in J.RT3-T3.5 (Jurkat derivative). (*B*) Discovery of the *FAM133B/CDK6* rearrangement by paired-end RNA-seq. The fusion junction was confirmed by RT-PCR (not shown) and Sanger sequencing. (*C*) Gene expression profiling reveals high-level expression of *CDK6* in J.RT3-T3.5 compared to other leukemia cell lines. Note that array probes mapped to the portion of CDK6 retained in the fusion. (*D*) Jurkat demonstrates marked sensitivity to the *CDK4/6* inhibitor PD0332991 (IC_50_ = 0.27 µM). K562, which expresses only wildtype CDK6, is used as a negative control cell line and shows minimal sensitivity to PD0332991 (IC_50_ = 5.9 µM).

Cyclin-dependent kinase 6 (*CDK6*) encodes a regulator of G_1_/S cell-cycle progression and has been found rearranged in B-cell lymphoma (*IGK/CDK6*), chronic lymphocytic leukemia (*IGL/CDK6, IGH/CDK6, IGK/CDK6*), and acute lymphoblastic leukemia (*CDK6/MLL*) [66]–[68]. DBA identified a focal DNA amplification disrupting *CDK6* in J.RT3-T3.5, a mutant TCR-negative Jurkat cell line derivative [69] (Figure 6A). To evaluate this further, we performed paired-end RNA-seq on Jurkat cells, which revealed a novel gene fusion, *FAM133B/CDK6* (Figure 6B). *CDK6* sits adjacent to *FAM133B* (an uncharacterized gene) at chr 7q21.2, and both genes are transcribed in the same direction. However, *CDK6* resides upstream of *FAM133B*; therefore the fusion might result from a tandem duplication event. The predicted fusion is in-frame, and juxtaposes 41 amino acids from the N-terminus of FAM133B to an N-terminally truncated CDK6. Analysis of publicly-available microarray data confirmed high-level expression of *CDK6* in J.RT3-T3.5, relative to other leukemia cell lines (Figure 6C; array probes mapped to the portion of CDK6 retained in the fusion). In addition, Jurkat cells exhibited marked sensitivity to the CDK4/6 inhibitor, PD0332991 (IC_50_ = 0.27 µM; Figure 6D).

### Rearrangement of *CLTC* and *VMP1* occurs in multiple cancer types

**Figure 7:**
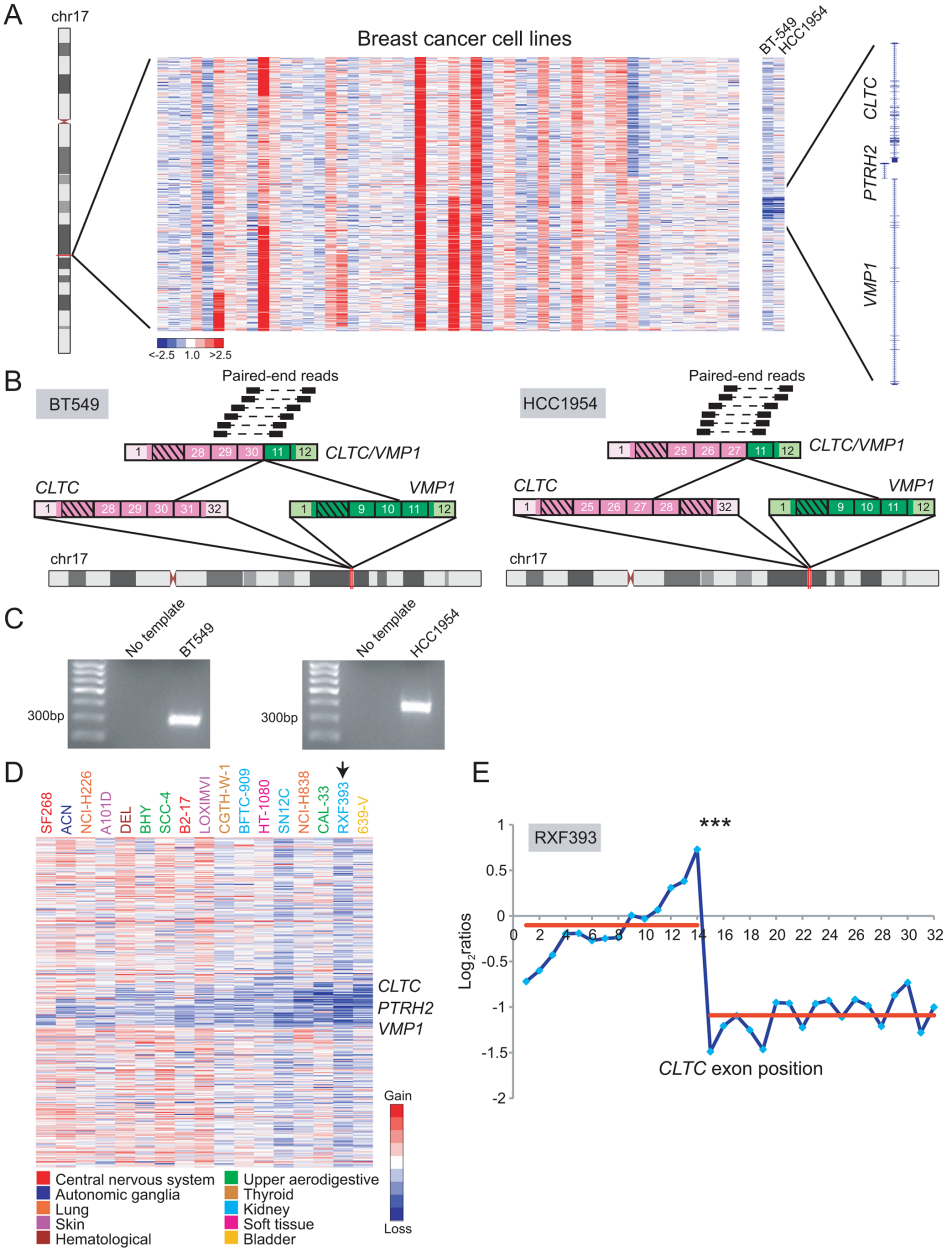
DBA discovery of recurrent rearrangements of *CLTC* and *VMP1* across diverse cancer types. (*A*) Heatmap depicting focal deletions between *CLTC* and *VMP1* in the breast cancer cell lines BT-549 and HCC1954. (*B*) Discovery of the recurrent *CLTC/VMP1* rearrangement in BT-549 (*left* panel) and HCC1954 (*right* panel) by paired-end RNA-seq. (*C*) RT-PCR verification of *CLTC/VMP1* fusion in BT-549 and HCC1954. (*D*) Heatmap depicting focal deletions disrupting *CLTC*, *PTRH2* and/or *VMP1* in various cancer types (see legend). (*E*) A renal cell carcinoma line, RXF393, was also profiled by exon microarray where an expression breakpoint was evident within *CLTC*. ***P<10^−9^ (Student's t-test).

Gene fusions involving clathrin heavy chain (*CLTC*) have been described in various leukemias (*CLTC/ALK*) and in renal cell carcinoma (*CLTC/TFE3*) [70]–[72]. DBA suggested that *CLTC* rearrangements might be more widespread in human malignancies (Table S2). Copy-number transitions within cytoband 17q23.1 occurred as focal deletions that involved three neighboring genes, *CLTC*, *PTRH2*, and *VMP1* (also called TMEM49). We selected to further evaluate two breast cancer cell lines, BT-549 and HCC1954, with deletions spanning *CLTC*-*VMP1* (Figure 7A). Paired-end RNA-seq revealed a distinct *CLTC/VMP1* fusion transcript in each sample (Figure 7B, 7C). Notably, both *CLTC/VMP1* fusions were predicted to be out of frame. A recent study also identified the *CLTC/VMP1* fusion in BT-549 [73]; our findings now demonstrate this to be a recurrent rearrangement in breast cancer.

A similar deletion pattern occurred in other malignancies, including glioblastoma, neuroblastoma, lung cancer, bladder cancer, thyroid cancer, melanoma, leukemia, and others (Figure 7D). Across all these samples, the minimum common region of deletion appeared to include only *PTRH2* and *VMP1*. One of the samples, renal cell carcinoma line RXF393, was also analyzed by RBA, where a candidate *CLTC* rearrangement was identified (Figure 7E).

### Novel cell line models for *EGFRVIII* and *FIP1L1/PDGFRA*

**Figure 8:**
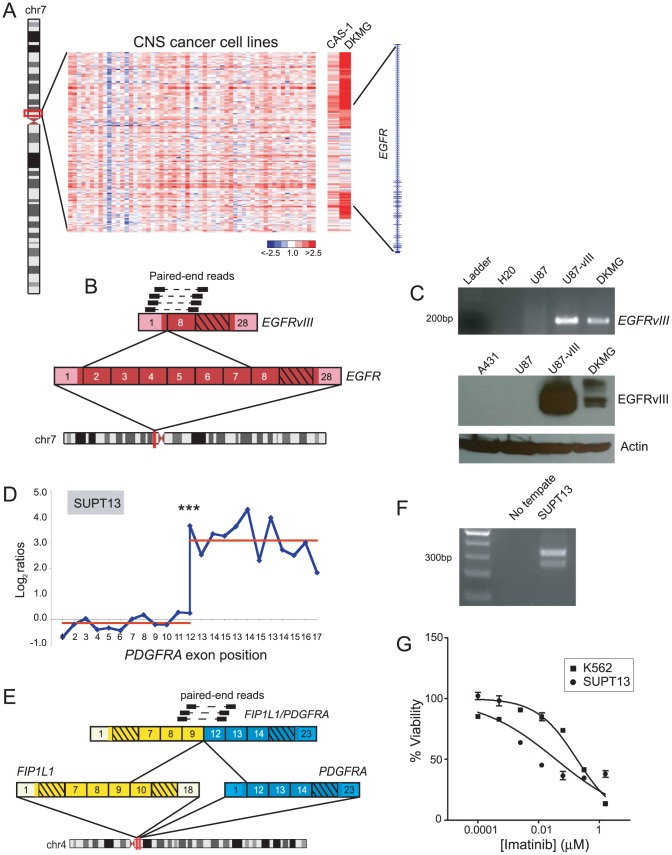
Discovery of new cell line models for the known rearrangements, *EGFRvIII* and *FIP1L1/PDGFRA*. (*A*) Heatmap depicting genomic breakpoints within *EGFR* in the glioblastoma cell lines, CAS-1 and DKMG. (*B*) Identification of *EGFRvIII* in DKMG cells by paired-end RNA-seq. Paired-end reads supporting the rearrangement are depicted. (*C*) Verification of *EGFRvIII* expression by RT-PCR (top panel) and Western blotting (bottom panel) in DKMG. RT-PCR was done using primers flanking the exon 1/exon 8 junction of *EGFRvIII*, and Western blotting was done using an antibody specific to the EGFRvIII isoform. Control samples include U87 glioblastoma cells without *EGFR* rearrangement, U87-vIII cells engineered to express exogenous *EGFRvIII*, and A431 epidermoid carcinoma cells with *EGFR* amplification. (*D*) RBA identification of expression-level breakpoint within *PDGFRA* in SUPT13 T-ALL cells. ***P<10^−11^ (Student's t-test). (*E*) RNA-seq identification of *FIP1L1/PDGFRA*. (*F*) RT-PCR validation of *FIP1L1/PDGFRA* expression in SUPT13. (*G*) SUPT13 cells are sensitive to imatinib (IC_50_ = 0.036 µM). K562 is a positive control CML cell line harboring BCR/ABL1 with known sensitivity to imatinib (IC_50_ = 0.18 µM).

In addition to discovering novel gene fusions, our breakpoint analysis approach proved useful for identifying new cell line models for known oncogenic rearrangements. In particular, DBA identified genomic breakpoints within epidermal growth factor receptor (*EGFR*) in two glioblastoma multiforme cell lines, DKMG and CAS-1 (Figure 8A). Approximately 20–30% of glioblastoma tumors harbor a constitutively active rearrangement of *EGFR*, called *EGFRvIII*, but glioblastoma derived cell lines typically lose *EGFR* amplification and EGFRvIII expression [74], [75]. Hence, studies of *EGFRvIII* have been hindered by the lack of suitable cell line models. Paired-end RNA-seq, followed by RT-PCR and Western blotting, revealed the expression of EGFRvIII in DKMG cells (Figure 8B, 8C). Further functional characterization of EGFRvIII in this cell line is described elsewhere [76].

DBA also identified DNA copy-number transitions within platelet-derived growth factor receptor alpha (*PDGFRA*) in glioblastoma (SNB19) and chronic eosinophilic leukemia (EOL-1) (Table S2). RBA identified a corroborating expression-level transition within *PDGFRA* in EOL-1 (Table S1, Figure S4C), and another in the T-ALL cell line SUPT13 (Figure 8D). The *FIP1L1/PDGFRA* fusion is a hallmark of chronic eosinophilic leukemia and has been studied extensively in EOL-1 cells [77], [78], but other cell line models are lacking. We performed paired-end RNA-seq on SUPT13 and found that it harbors *FIP1L1/PDGFRA* (Figure 8E, 8F), albeit with a distinct gene fusion junction from EOL-1. Notably, SUPT13 cells also demonstrated marked sensitivity to the PDGFR inhibitor, imatinib mesylate (IC_50_ = 0.036 µM) (Figure 8G). Thus, SUPT13 represents a new cell line model for studies of this known gene fusion.

## Discussion

Here, we have described the development and implementation of a breakpoint analysis pipeline for cancer gene fusion discovery, which we applied to a large collection of nearly 1,000 cancer samples. We discovered novel gene rearrangements in diverse human cancer types, including fusions of *ROS1*, *SLC1A2*, *RAF1*, *EWSR1*, *CDK6*, and *CLTC*.

The *ROS1* rearrangement (*CEP85L/ROS1*), to our knowledge, represents the first gene fusion described in angiosarcoma. By FISH analysis, *ROS1* rearrangements appear to be infrequent in angiosarcoma (and in another endothelial-derived tumor, epithelioid hemangioendothelioma). Nevertheless, the finding of elevated *ROS1* expression in angiosarcomas, relative to other sarcoma subtypes, suggests that ROS1 might play a broader role in angiosarcoma pathogenesis. Angiosarcoma is an aggressive sarcoma subtype, with an overall 5-year survival rate of approximately 35% [48]. Locally recurrent and metastatic tumors are generally chemoresistant. As a tyrosine kinase, ROS1 represents a potential new therapeutic opportunity. In this regard, we note that ROS1 tyrosine kinase is sensitive to the existing ALK small-molecule inhibitor, crizotinib, and indeed a single patient's non-small cell lung cancer harboring a *ROS1* fusion was found to be responsive [79]. Intriguingly, single nucleotide polymorphism (SNP) variants in *ROS1* have been associated with increased risk of vascular diseases, including coronary artery disease and stroke [80], [81]. These reports possibly suggest an even broader link between ROS1 and endothelial cell pathobiology.

Our findings also demonstrate a more widespread role of *SLC1A2* rearrangements in human malignancies. We show that in addition to *CD44/SLC1A2* in gastric cancer, *SLC1A2* is involved in a novel but analogous gene fusion, *APIP/SLC1A2*, in colon cancer. Both of these rearrangements are predicted to overexpress the identical N-terminally truncated SLC1A2 protein, a functioning glutamate transporter. Notably, while most oncogenic gene fusions encode protein kinases and transcription factors [3], [40], [82], *SLC1A2* fusions appear to define a new class of rearrangement targeting metabolism-related genes [27]. Indeed, altered cell metabolism, is increasingly recognized as a primary driver of human cancer [83]. *SLC1A2* fusions therefore also represent potential therapeutic targets in gastric and now colon cancer. Pharmacological inhibitors of several transporter proteins have been developed [84]. However, as glutamate is a major excitatory neurotransmitter of the central nervous system, a monoclonal antibody targeting *SLC1A2* might provide an alternative anti-cancer agent, where the larger size would limit crossing the blood-brain barrier.

Our analysis also uncovered *RAF1* rearrangements in pancreatic cancer and in anaplastic astrocytoma. To our knowledge, these are the first fusion genes reported in either cancer type. On a cautionary note, most pilocytic astrocytomas (a distinct diagnosis, but related to anaplastic astrocytoma) carry *RAF1* or *BRAF* rearrangements; thus it is possible that the D538-MG cell line (harboring *BCL6/RAF1*) was actually derived from a misdiagnosed pilocytic astrocytoma. Regardless, *BCL6/RAF1* constitutes a novel *RAF1*-partnered fusion. Our findings extend the spectrum of cancer types harboring RAF kinase rearrangements, and underscore the importance of the RAS-RAF-MAPK signaling pathway in these additional malignancies. In pancreatic cancer, pathway activation typically occurs by mutation of *KRAS*, but in uncommon *KRAS*-wildtype tumors, RAF kinase fusions may provide an alternative route. Though RAF kinase fusions are uncommon, they nonetheless have therapeutic implications for this deadly malignancy. Several RAF kinase and MAP kinase pathway inhibitors are now in clinical trials for various cancer types [85], [86].

Breakpoint analysis also identified a novel *EWSR1/CREM* fusion in melanoma (CHL-1). Several “singleton” gene fusions have been reported in melanoma, but it is unclear whether any of these rearrangements have oncogenic properties [20]. In addition, Palanisamy *et al.* found rearrangements of RAF kinase genomic loci by FISH in rare cases of melanoma, but no specific RAF kinase gene fusion was identified [17]. Thus, *EWSR1/CREM* potentially represents the first oncogenic gene fusion discovered to date in melanoma. *EWSR1* rearrangements in Ewing's sarcoma have recently been shown to confer sensitivity to PARP-1 inhibition [87]. Advanced melanomas carry a poor prognosis and are generally unresponsive to anti-cancer medications or rapidly acquire resistance to these agents. The potential role of *EWSR1/CREM* as a marker for PARP-1 inhibitor sensitivity should be further explored.

Our discovery of a *CDK6* fusion in T-ALL also carries important pathobiologic and clinical implications. In knockout studies in mice, CDK6 was recently shown to play a role in thymocyte development and tumorigenesis [88]. Thus, it is plausible that the CDK6 rearrangement drives deregulated CDK6 expression and T-cell derived leukemia. Our findings provide a rationale for preclinical testing and clinical trials using existing CDK6 inhibitors (e.g. PD0332991).

Lastly our breakpoint analysis uncovered recurrent deletions and rearrangements of the *CLTC-PTRH2-VMP1* locus, evident in diverse tumor types, including glioblastoma, neuroblastoma, lung cancer, breast cancer, bladder cancer, thyroid cancer, melanoma, and leukemias. In breast cancer, we discovered two *CLTC-VMP1* fusions; however, both were out-of-frame. These findings are most consistent with one or more of the three genes at this locus functioning as a tumor suppressor in multiple tumor types. Notably, *PTRH2*, the centrally residing gene at this locus, encodes a mitochondrial protein that induces apoptosis through interactions with the small Groucho family transcriptional regulator, AES, consistent with a tumor suppressive function [89].

In the current study, we performed pharmacologic inhibition and RNAi knockdown experiments to functionally characterize several gene fusions, and we performed FISH to assess recurrence. The results of these experiments highlight the pathogenic roles of these alterations in their corresponding cancer types. However, not all rearrangements were fully characterized. In particular, we were unable to culture D538-MG cells, and so we did not perform experiments to assess the function of *BCL6/RAF1*. In addition, we were unable to efficiently transfect the suspension cell line SNU-C1 with siRNAs targeting *APIP/SLC1A2*. While the structures of these alterations strongly support oncogenic roles, further experiments must be undertaken to fully characterize their function. Additional FISH and RT-PCR experiments are also planned to further assess rearrangement frequencies for several gene fusions.

By our novel discoveries, we demonstrate that breakpoint analysis provides a powerful approach for gene fusion discovery. While our opportunities to integrate RBA and DBA were limited (due to the small overlap of samples), we expect that candidates identified by both methods would be further enriched for valid fusions. There exist now publicly-available microarray data for many thousands of cancer samples [29], [30], [90] which can be mined by breakpoint analysis. In particular, recurrent gene fusions appear to occur at low frequency in many cancer types, and therefore these existing very large sample sets should empower their discovery. While here we have applied breakpoint analysis to discover rearrangements of known cancer genes as part of novel fusions and in novel cancer types, our approach should be extendable to discover pathogenic fusion genes not previously linked to malignancy.

In summary, breakpoint analysis uncovered several novel gene rearrangements spanning multiple human cancer types. We identified new gene fusions involving *ROS1*, *SLC1A2*, *RAF1*, *EWSR1*, *CDK6*, and *CLTC*, some occurring in cancer types not previously known to harbor fusions. Several of these fusions represent druggable targets or potential markers for sensitivity to specific anti-cancer treatments with therapeutic implications for the corresponding cancer types. Importantly, such multi-tumor rearrangements support the notion that tumors might be better classified by their underlying molecular alterations, rather than their tissue of origin.

## Materials and Methods

### Exon microarray expression datasets

For RBA, we mined data from 76 publicly-available exon-resolution expression arrays, done on Affymetrix Human Exon 1.0 ST microarrays, and including 17 T-ALL (GSE9342) cell lines and all 59 of the NCI-60 cancer cell lines (GSE29682) [33]. Affymetrix Expression Console software was used to extract normalized log_2_ ratios from raw data files using the RMA-sketch algorithm from Affymetrix's Power Tools package. Exon log_2_ ratios were then mean centered across the array set. In addition, we profiled 16 cancer samples on a custom Agilent 8×15K microarray that contained 325 genes previously known to be involved in oncogenic rearrangements. The sample set included 8 positive control samples harboring known rearrangements, used to optimize our analysis pipeline, as well as 8 sarcoma specimens representing sarcoma subtypes where gene fusions had not yet been described. For the custom arrays, sample labeling was done using the Fairplay III Microarray Labeling Kit (Agilent). Briefly, 10 µg of sample total RNA and 1 µg of reference mRNA (pooled from 11 diverse cell lines; [91]) were differentially labeled with Cy5 and Cy3, respectively, and co-hybridized to the microarray. Following overnight hybridization and washing, arrays were imaged using Agilent's High-Resolution C Scanner. Normalized fluorescence ratios were extracted using Agilent Feature Extraction Software, and values were mean centered across samples.

### Array CGH datasets

For DBA, we mined data from 812 CGH/SNP arrays, representing cancer cell lines derived from 29 distinct tissues, from the Wellcome Trust Sanger Institute's Cancer Genome Project [31]. These cell lines were profiled on Affymetrix SNP 6.0 microarrays containing 1.8 million genetic markers including more than 946,000 probes for the detection of copy number variation. Affymetrix Genotyping Console software was used to extract probeset intensities from raw data files using the regional GC correction configuration for Copy Number/LOH analysis and default settings. Intensities were normalized against a HapMap 270 normal reference dataset, and log_2_ ratios were analyzed for genomic breakpoints. In addition, we analyzed a pancreatic cancer dataset generated by our laboratory, consisting of 22 pancreatic cancer cell lines and 48 early-passage xenografts [34]. These samples were profiled on Agilent 244K CGH arrays and normalized log_2_ ratios were obtained as described [34].

### RNA breakpoint analysis

RBA was implemented using custom C# scripts. The RBA algorithm is based on a “walking” Student's t-test, which for every exon-exon junction along the transcript compares expression levels of all proximal *vs.* distal exons (see Figure S2A). The algorithm was applied to all annotated genes and subsequently filtered for candidate expression breakpoints disrupting genes previously identified in oncogenic rearrangements, as defined by the Cancer Gene Census [40]. The Cancer Gene Census was downloaded in November 2011 from the Wellcome Trust Sanger Institute (http://www.sanger.ac.uk/genetics/CGP/Census/). We filtered this list to exclude known common fragile sites, as well as non-oncogenic fusion partners such as those involved in rearrangements with MLL and the 5′ partners of tyrosine kinase fusions, with the exception of promiscuously rearranged genes (i.e. those involved in multiple distinct gene fusions). We also included *SLC1A2*, which has recently been discovered to form oncogenic gene fusions in gastric cancer [27], but had not yet been added to the census. The resulting filtered list included 306 genes. Statistical significance (*P*<0.05) was determined using a Bonferroni correction to adjust for multiple t-tests. Specifically, 3,218 t-tests were performed for each Affymetrix microarray experiment, with significance corresponding to an uncorrected *P* = 1.55×10^−5^, and 1,807 t-tests were performed for each custom microarray experiment, with significance corresponding to an uncorrected *P* = 2.77×10^−5^. Positive hits were defined as genes with *P*-values dipping below the significance threshold during the walking t-test. We only included expression breakpoints with directional orientation (i.e. being the 5′ or 3′ partner) corresponding to that of known rearrangements involving a given gene.

### DNA breakpoint analysis

DBA was done using a combination of publicly available software and custom C# scripts. Copy number alterations (CNAs) were initially determined from normalized log_2_ ratios using the fused lasso algorithm (FDR 1%) [92]. We then used a custom algorithm to better define the boundaries of each CNA (thereby minimizing overcalled transitions), which we termed “copy number smoothing.” Copy number smoothing was applied to each chromosome of each profiled sample, where each iteration begins by identifying the upper (5′) boundary of the subsequent candidate “well-defined” CNA called by fused lasso. A well-defined CNA was defined by an average |log_2_| ratio greater than or equal to an adjustable threshold (here set to 0.3) and a minimum length of at least 50 probe sets. Adjusting the log_2_ ratio threshold affected the number of nominated gene fusions. We empirically chose a threshold that enabled detection of many known gene fusions, such as *EWSR1/FLI1* in Ewing's sarcoma, while minimizing false positives (Table S4). For high-level CNAs, defined by |log_2_| ratio greater than or equal to 1.0, we permitted a minimum length of only 10 probe sets, because we observed that focal high-level copy number transitions often characterized known rearrangements, e.g. *BCR/ABL1* (K562), *MLL* (OCI-AML2), *EWSR1/FLI1* (CADO-ES1, EW18), and *CD44/SLC1A2* (SNU-16). After finding this upper boundary, the algorithm walks down the CNA to identify its lower (3′) boundary. The lower boundary is defined as either reaching the end of the chromosome or finding the position where 95% of the subsequent 100 ratios meet any one of the following criteria: (1) copy number neutral (log_2_ ratio = 0); (2) change in the log_2_ ratio sign, i.e. from (−) to (+) or vice versa; or (3) average |log_2_| ratio that changes by an adjustable threshold (here set to 0.3). For high-level CNAs, 95% of only the next 50 ratios are evaluated using these criteria. After finding the upper and lower boundaries of a given CNA, its average value is determined. A second custom C# script then mines the CNAs for those that disrupt annotated genes. These candidates were further filtered to include only the subset disrupting Cancer Gene Census genes. We also prioritized those breakpoints where the directional orientation of the copy number transition corresponded to that of known rearrangements of the particular gene. For example, breakpoints disrupting *ABL1* kinase must comprise either amplification of the 3′ end or deletion of the 5′ end of the gene, since *ABL1* is the 3′ partner in known oncogenic rearrangements such as *BCR/ABL1*.

### Gene expression datasets and supervised analysis

To analyze expression levels, we also mined microarray gene-expression data including 76 leukemia cell lines profiled on Affymetrix Human Genome U133 Plus 2.0 microarrays from the National Cancer Institute's caArray database (https://array.nci.nih.gov/caarray/project/woost-00041), 67 sarcoma specimens profiled on cDNA microarrays printed at Stanford [53]–[55], 136 normal solid tissue samples profiled on Affymetrix Human Genome U133A microarrays [56], [57], 67 colon cancer cell lines profiled on the Rosetta/Merck Human RSTA Custom Affymetrix 2.0 microarrays [58], and a subset of the gene-expression profiling data (Affymetrix U133 plus 2.0 arrays) from the Cancer Cell Line Encyclopedia (CCLE) [93]. An angiosarcoma gene-expression signature was defined as previously described [94], as those genes meeting the following criteria: (1) gene expression correlated (Pearson correlation |R|≥*0.5*) with angiosarcoma subtype considered as a binary variable; (2) gene expression significantly altered in angiosarcoma samples (two-tailed Student's t-test, P<0.001); and (3) ≥2-fold difference in average expression between angiosarcomas and other sarcoma specimens. To estimate a FDR (i.e. fraction of genes falsely called significant), we compared our results to those obtained from 1,000 trials with class labels (i.e. angiosarcoma versus other sarcomas) randomly permuted.

### Cell lines and tissues

Cell lines SNU-C1, BT-549, HCC1954, SK-ES-1, A-172, K562, A431, CHL-1, SH-4, VCaP and J-RT3-T3-5 were obtained from the American Type Culture Collection. DK-MG, SU-DHL-1, and EOL-1 were obtained from the German Collection of Microorganisms and Cell Cultures (DSMZ). ONS-76 was obtained from the Health Science Research Resources Bank (HSRRB, Tokyo, Japan). The remaining cell lines were kind gifts from different research laboratories including D-538MG (Dr. Darell Bigner, Duke University), PL5 (Dr. Anirban Maitra, Johns Hopkins University), and SUPT-13 (Dr. Michael Cleary, Stanford University). Cell lines were propagated in RPMI-1640 supplemented with 10% fetal bovine serum (FBS), except for VCaP (DMEM with 10% FBS), D-538MG (Richter's zinc option medium (Invitrogen) with 10% FBS), and SK-ES-1 (McCoy's 5A Medium with 15% FBS). Cells were harvested at 80% confluency. Freshly-frozen cancer specimens were obtained from the Stanford Tissue Bank, collected with IRB approval and patient informed consent. Total RNA from tumors and cell lines was isolated using the RNeasy Mini Kit (Qiagen).

### RACE–PCR

Rapid extension of cDNA ends (RACE), done using the GeneRacer Kit (Invitrogen), was used to identify the 5′ fusion partner of *ROS1* in the AS1 specimen (prior to our development of an RNA-seq pipeline). In brief, 5 µg of total RNA from AS1 was treated with calf intestinal phosphatase to remove the 5′ phosphate group from truncated or non-mRNA molecules. Next, the sample was treated with tobacco acid pyrophosphatase to remove the 5′ cap structure from full length mRNA, to create a free 5′ phosphate group for subsequent adapter ligation. These molecules were ligated to the GeneRacer RNA oligo. Random primers and SuperScript III were then used to produce the RACE ready cDNA. The GeneRacer 5′ primer served as the forward primer and a custom primer designed within exon 35 of *ROS1* (AGTTGGCTGAGCTGCGAGGTCTG) was used as a reverse primer. RACE PCR reactions were resolved on a 1% agarose gel. A 600 bp band was purified and Sanger sequenced.

### Paired-end library preparation for Illumina sequencing

Paired-end transcriptome sequencing (RNA-seq) was done to discover the identity of the fusion partner of candidate fusion genes. Adapter-ligated cDNA libraries were prepared using the mRNA Seq-8 Sample Prep Kit (Illumina). Briefly, mRNA was isolated and purified from 1 to 10 µg of total RNA using Sera-Mag Magnetic Oligo(dT) Beads. mRNA was subsequently fragmented at 94°C in a fragmentation buffer and converted to single stranded cDNA using SuperScript II reverse transcriptase (Invitrogen). Subsequently second-strand cDNA synthesis was performed using E. coli DNA polymerase I (Invitrogen). Double stranded cDNA was end repaired using T4 DNA polymerase and T4 polynucleotide kinase, and then monoadenylated using Klenow DNA polymerase I. Adapter sequences were ligated to library molecules using T4 DNA ligase. Library fragments were then size selected (300–400 bp) on a 2% agarose gel and then purified using the QIAquick Gel Extraction Kit (Qiagen). Purified cDNA fragments were enriched with 15 PCR cycles using Phusion DNA Polymerase and provided buffers. Libraries were again electrophoresed and then gel purified using the Qiaquick Minelute Gel Purification Kit (Qiagen). Adapter ligated cDNA libraries were quantified with the Agilent DNA 1000 kit on the Agilent 2100 Bioanalyzer. Libraries were sequenced on either the Genome Analyzer II or HiSeq 2000 instruments (Illumina).

### Paired-end gene fusion discovery pipeline

Mate-paired RNA-seq reads were mapped to the human genome (hg18) and the RefSeq transcriptome allowing up to 2 mismatches, using Efficient Alignment of Nucleotide Databases (ELAND). For a given sample and its corresponding candidate gene fusion, a custom C# script was used to extract all mate pairs with one read mapping to the candidate rearranged gene and the other read mapping to a different genomic locus. The mapping position(s) of the paired read were used to nominate candidate gene fusion partners. A series of filters was then applied to distinguish nominated rearrangements from artifacts arising during library construction. Specifically, the median predicted distance between paired reads was required to be between 100 and 400 nts. Nominated fusions involving genes located adjacent to one another and oriented in the same direction on the chromosome (i.e. likely “readthrough” transcripts) were filtered out. In addition, a second C# script was designed to screen for mate pairs with single reads spanning potential exon-exon fusion junctions (chimeric reads) of nominated gene fusions. Briefly, we screened for mate pairs with a single read mapping to either gene in a nominated gene fusion and with a second non-mapping read. The script attempted to align these non-mapping reads to various exon-exon combinations from the two genes involved in the nominated rearrangement. Identified chimeric reads were merged with the other mate pairs supporting the nominated gene fusion. Nominated rearrangements with less than two supporting mate pairs were filtered out and candidates were validated by RT-PCR followed by Sanger sequencing.

### RT–PCR validation of fusions

Specimen RNAs were reverse transcribed using SuperScript III reverse transcriptase with random hexamers (Invitrogen). Primers used for RT-PCR gene fusion validation are listed in Table S5. PCR reactions were resolved on 1% agarose TAE gels, and bands were purified and Sanger-sequenced to verify predicted fusion junctions. For validation of the EGFRvIII gene product [95], RT-PCR was performed using 200 ng of total RNA and the One-Step RT-PCR kit (Qiagen). Reverse transcription was done at 52°C for 45 minutes, 60°C for 1 minute, and 52°C for 30 minutes, followed by enzyme inactivation and hot-start PCR at 95°C for 15 minutes. Denaturation, annealing, and extension were done at 93°C, 60°C, and 72°C, for 30 seconds, 1 minute, and 45 seconds, respectively, for a total of 40 cycles, with a final extension period at 72°C for 10 minutes. Reaction products were electrophoresed in 2% agarose gels and stained with SYBR Green.

### Break-apart FISH assays

Probe labeling and FISH were performed using Vysis/Abbott Molecular reagents and protocols. Locus-specific BACs encompassing *ROS1* (CTD-2174H19 telomeric, RP11-605K7 centromeric), *RAF1* (RP11-586C12 telomeric, RP11-767C1 centromeric), and *BRAF* (RP11-364M15 telomeric, RP11-597I24 centromeric) were labeled with Cy5-dUTP (telomeric probes) or Cy3-dUTP (centromeric probes). Chromosomal locations of BACs were first validated using normal metaphase slides. Fluorescently labeled probes interrogating *ROS1* were hybridized to TMAs containing 280 sarcoma and soft tissue tumor specimens. Probes interrogating *RAF1* and *BRAF* were hybridized to TMAs containing 104 evaluable pancreatic cancer cases. Slides were counterstained with DAPI, and imaged using an Olympus BX51 fluorescence microscope with Applied Imaging Ariol 3.0 software. Rearrangement was defined by physical separation of the red and green FISH signals, or loss of the red or green FISH signal, in at least 25% of tumor nuclei.

### siRNA transfections

On-TARGETplus siRNAs targeting *RAF1* and *CREM*, as well as a non-targeting control siRNA pool (ON-TARGETplus siCONTROL Non-targeting Pool), were obtained from Dharmacon. Cell lines were seeded at a density of 75,000–150,000 cells per 6-well plate well and transfected using Lipofectamine 2000 reagent (Invitrogen). Cells were transfected with a final concentration of 25 nM siRNA for 16 hours in Opti-Mem (GIBCO), which was subsequently replaced with complete growth media (RPMI-1640 with 10% FBS).

### Q–RT–PCR and Western blots

Q–RT–PCR was performed using Assay-on-Demand TaqMan probes and reagents (Applied Biosystems). A custom primer set encompassing the EWSR1/CREM gene fusion junction was designed to interrogate expression of the gene fusion in CHL-1 (GCCAACAGAGCAGCAGCTA, GGATCTGGTAAGTTGGCATGTCA). Western blots were done on whole cell lysates, using the following primary antibodies: anti-RAF1 rabbit polyclonal (1∶200; Cell Signaling); anti-EGFRvIII (1∶1000, [95]); anti-GAPDH rabbit polyclonal antibody (1∶5000; Santa Cruz Biotechnology); β-actin (1∶10,000; Chemicon).

### Cell proliferation, invasion, and senescence assays

Cell viability/proliferation was quantified by colorimetry associated with cleavage of the tetrazolium salt, WST-1(Roche). Briefly, 10% WST-1 reagent was added to cells at 1, 3, and 5 days post siRNA transfection and then incubated at 37°C for 30 minutes. Absorbance was measured at 450 nm with reference to 650 nm using a Spectra Max 190 plate reader (Molecular Devices). Invasion was quantified by the Boyden chamber assay (BD Biosciences). Briefly, siRNA transfected cells were plated at a density of 20,000 cells per 24-well insert. A chemotactic gradient of 1% to 10% FBS was established, and cells were fixed and stained with crystal violet 48 hours post transfection. Cells traversing the membrane were counted. Senescence was assessed 72 hours post transfection using the Senescence β-Galactosidase Staining Kit (Cell Signaling) according to the manufacturer's instructions. Cells were washed with 1× PBS and then treated with a fixative solution. Cells were then stained for β-Galactosidase and counted. All assays were performed as biological triplicate, and mean values together with SDs are reported. All experiments were reproduced at least once.

### Gleevec, sorafenib, and PD0332991 treatment

Gleevec and sorafenib were obtained from LC Laboratories (Woburn, MA) and PD0332991 was obtained from Selleck Chemicals (Houston, TX). Agents were reconstituted in DMSO and used at the indicated concentrations. IC_50_ values were determined by fitting sigmoidal (four-parameter logistic) curves with Prism 4.0 software (GraphPad).

### Data access

All microarray and short-read sequencing data have been deposited in the NCBI Gene Expression Omnibus and Short Read Archive under the accession no. GSE45137.

## Supporting Information

Figure S1 Datasets and cancer types included for breakpoint analysis. Pie-charts of cancer type representation for (*A*) the 92 exon microarray profiles included in RBA, and (*B*) the 882 aCGH profiles included in DBA. Cancer types indicated in descending order of sample size, clockwise from 12 o'clock.
(PDF)

Figure S2 RBA for discovery of gene fusions. (*A*) Depiction of the walking t-test algorithm, illustrated for *NOTCH1* in SUPT-1 cells (known to carry a *TCRB*/*NOTCH1* rearrangement). At each exon-exon junction along the transcript, a Student's t-test is performed comparing the expression levels (green line, *above*) of exons proximal and distal to that junction. *P*-values are plotted (blue line, *below*) and a positive hit is recorded if a *P*-value drops below a significance threshold defined by Bonferroni adjustment (red dashed line). The minimum *P*-value corresponds to the predicted breakpoint for the gene fusion. (*B*) Distribution of walking t-statistics for all samples analyzed by RBA. Note that known gene fusions (red arrows) tend to have “outlier” *P*-values compared to most transcripts. (*C*) Distribution of the 54 candidate rearrangements nominated by RBA across cancer types.
(PDF)

Figure S3 DBA pipeline for gene fusion discovery. (*A*) DBA pipeline. Fused lasso (FDR 1%) is used initially to call copy number alterations (CNAs). We found that fused lasso tends to overcall transitions (breakpoints) in copy number status. Thus, we applied a custom method, termed “copy number smoothing” to identify well-defined CNAs and to better determine their upper and lower boundaries. Breakpoints are then screened for those disrupting Cancer Gene Census genes. In this depiction, a breakpoint disrupting *PDGFRA* corresponds to the *FIP1L1/PDGFRA* rearrangement in the EOL-1 leukemia cell line. (*B*) Distribution of the 144 intragenic breakpoints identified by DBA across cancer types.
(PDF)

Figure S4 RBA rediscovery of known gene fusions in various cancers. Exonic expression breakpoints representing known gene fusions including (*A*) *BCR/ABL1* in K562 (CML), (*B*) *NPM1/ALK* in SUDHL-1 (ALCL), (*C*) *FIP1L1/PDGFRA* in EOL-1 (eosinophilic leukemia), (*D*) *CCDC6/RET* in TPC-1 (thyroid cancer), (*E*) *NUP214/ABL1* in ALL-SIL, (*F*) *EWSR1/FLI1* in SKES-1 (Ewing sarcoma).
(PDF)

Figure S5 DBA rediscovery of known gene fusions in various cancers. (*A*) Heatmaps depicting identified intragenic breakpoints disrupting (*A*) *FLI1* in four Ewing's sarcoma cell lines (*EWSR1/FLI1*), (*B*) *ABL1* in seven CML (*BCR/ABL1*) and T-ALL (*NUP214/ABL1*) cell lines, and (*C*) *ROS1* in glioblastoma cell line U-118MG (*GOPC/ROS1*). Samples without rearrangement are also depicted for comparison.
(PDF)

Table S1 Candidate rearrangements nominated by RBA.
(XLS)

Table S2 Candidate rearrangements nominated by DBA.
(XLS)

Table S3 Sarcoma subtypes included on TMA.
(XLS)

Table S4 Affect of filtering parameters on DBA analysis of bone cancer cell lines.
(XLS)

Table S5 RT–PCR primers (for validation of candidate fusions).
(XLS)
