# Supplementary figures and images for: Breakpoint Analysis of Transcriptional and Genomic Profiles Uncovers Novel Gene Fusions Spanning Multiple Human Cancer Types
Source: PLoS Genet. 2013 Apr 25;9(4):e1003464. doi: 10.1371/journal.pgen.1003464 (PMC3636093; doi:10.1371/journal.pgen.1003464)

Figure S1

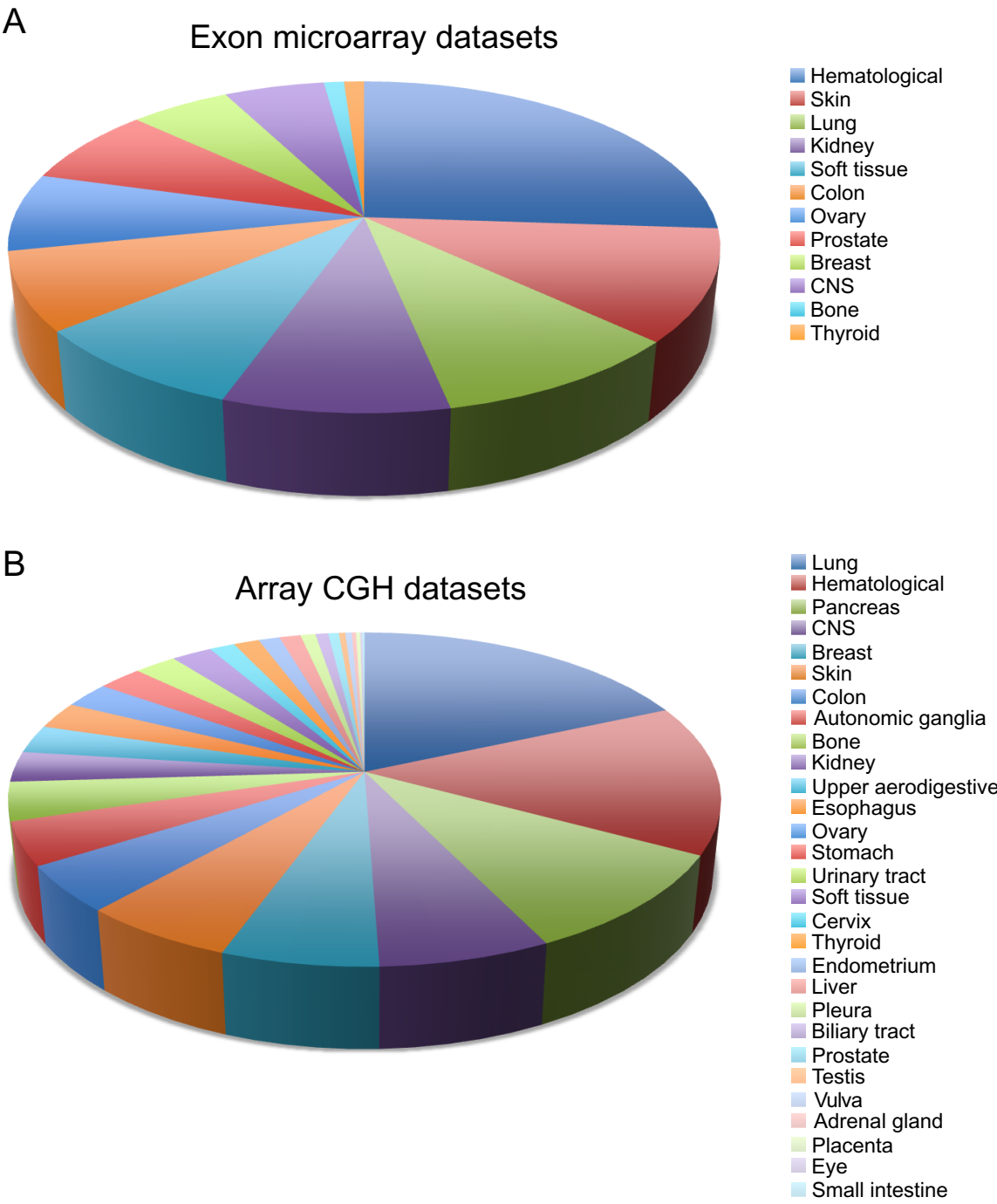

Supplement: Figure S1 — Datasets and cancer types included for breakpoint analysis. Pie-charts of cancer type representation for (A) the 92 exon microarray profiles included in RBA, and (B) the 882 aCGH profiles included in DBA. Cancer types indicated in descending order of sample size, clockwise from 12 o'clock. (PDF) [file pgen.1003464.s001.pdf]

Figure S2

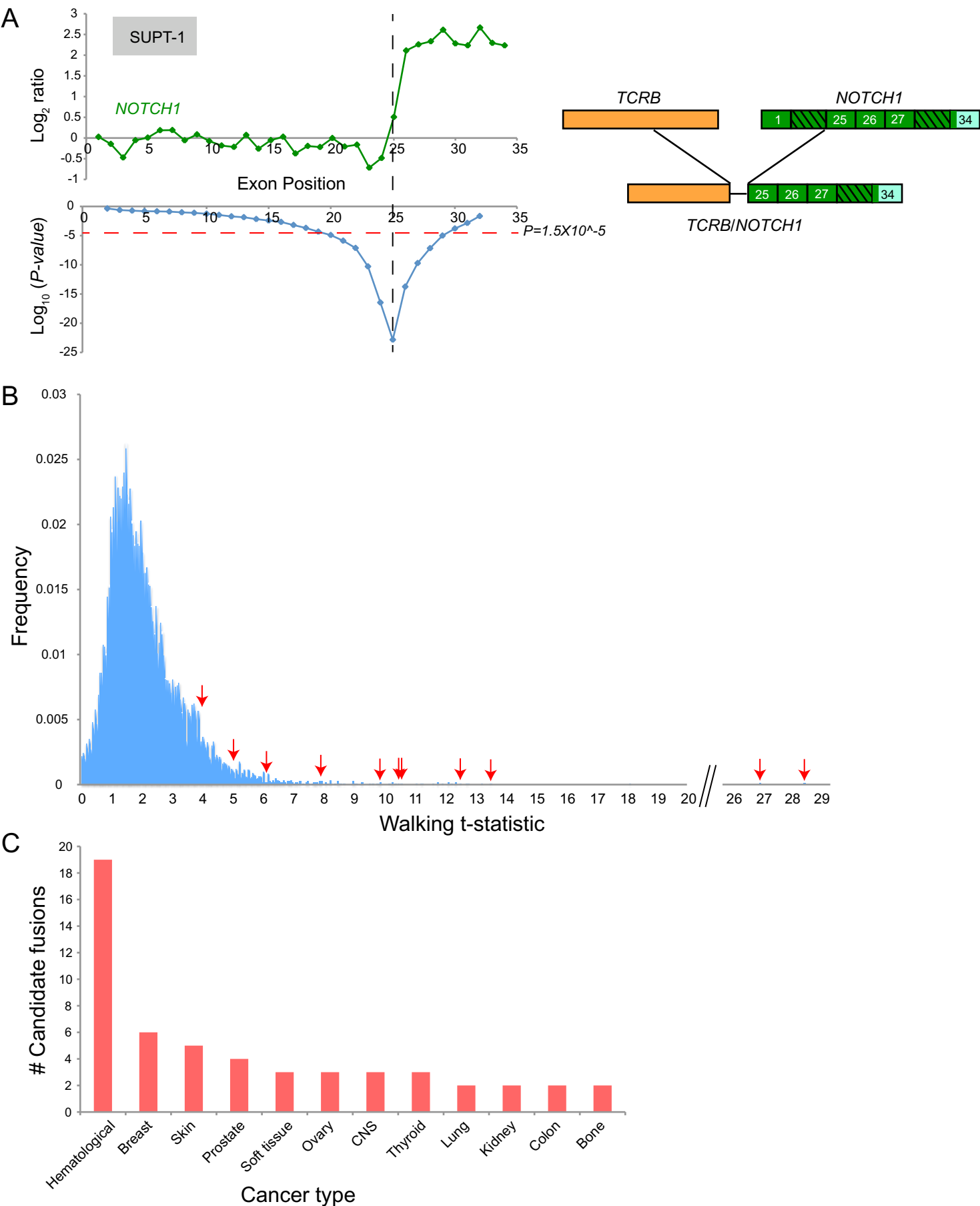

Supplement: Figure S2 — RBA for discovery of gene fusions. (A) Depiction of the walking t-test algorithm, illustrated for NOTCH1 in SUPT-1 cells (known to carry a TCRB/NOTCH1 rearrangement). At each exon-exon junction along the transcript, a Student's t-test is performed comparing the expression levels (green line, above) of exons proximal and distal to that junction. P-values are plotted (blue line, below) and a positive hit is recorded if a P-value drops below a significance threshold defined by Bonferroni adjustment (red dashed line). The minimum P-value corresponds to the predicted breakpoint for the gene fusion. (B) Distribution of walking t-statistics for all samples analyzed by RBA. Note that known gene fusions (red arrows) tend to have “outlier” P-values compared to most transcripts. (C) Distribution of the 54 candidate rearrangements nominated by RBA across cancer types. (PDF) [file pgen.1003464.s002.pdf]

Figure S3

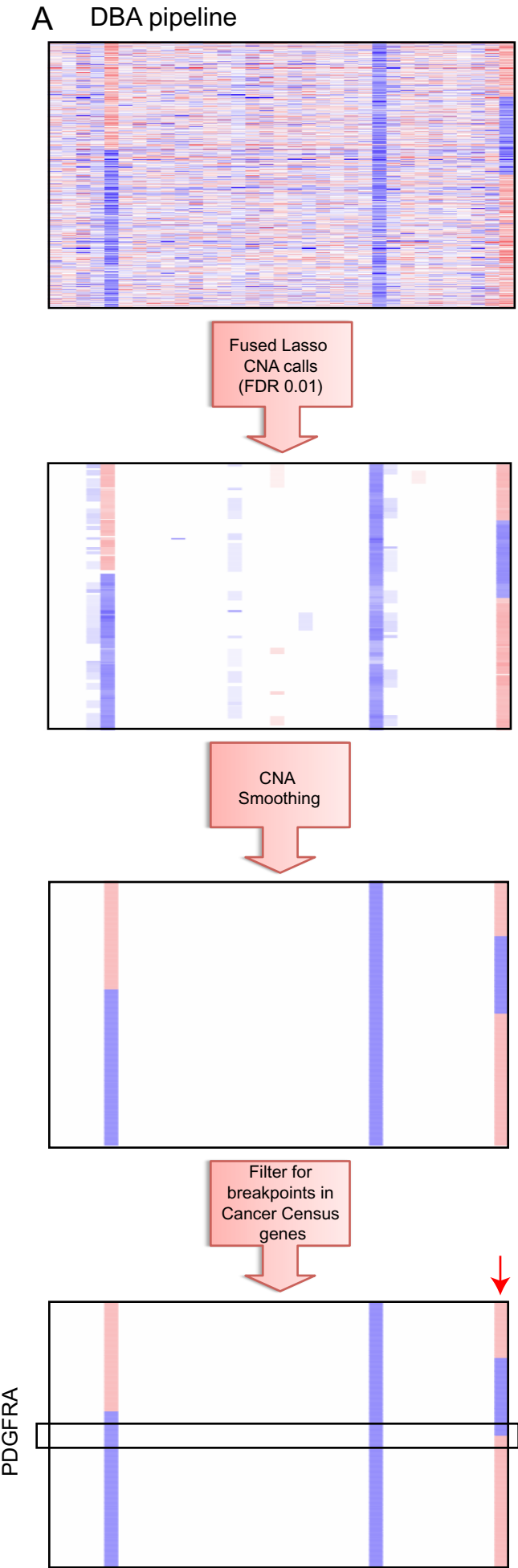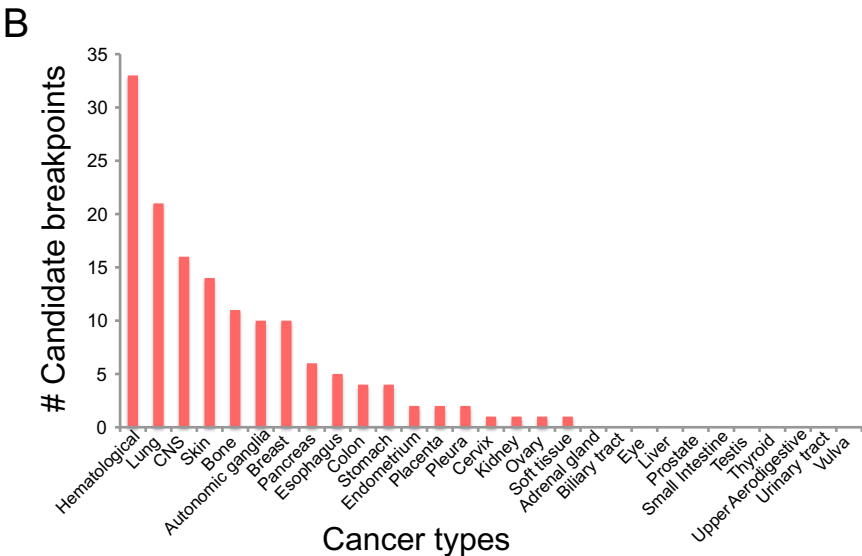

Supplement: Figure S3 — DBA pipeline for gene fusion discovery. (A) DBA pipeline. Fused lasso (FDR 1%) is used initially to call copy number alterations (CNAs). We found that fused lasso tends to overcall transitions (breakpoints) in copy number status. Thus, we applied a custom method, termed “copy number smoothing” to identify well-defined CNAs and to better determine their upper and lower boundaries. Breakpoints are then screened for those disrupting Cancer Gene Census genes. In this depiction, a breakpoint disrupting PDGFRA corresponds to the FIP1L1/PDGFRA rearrangement in the EOL-1 leukemia cell line. (B) Distribution of the 144 intragenic breakpoints identified by DBA across cancer types. (PDF) [file pgen.1003464.s003.pdf]

# Figure S4

A

K562

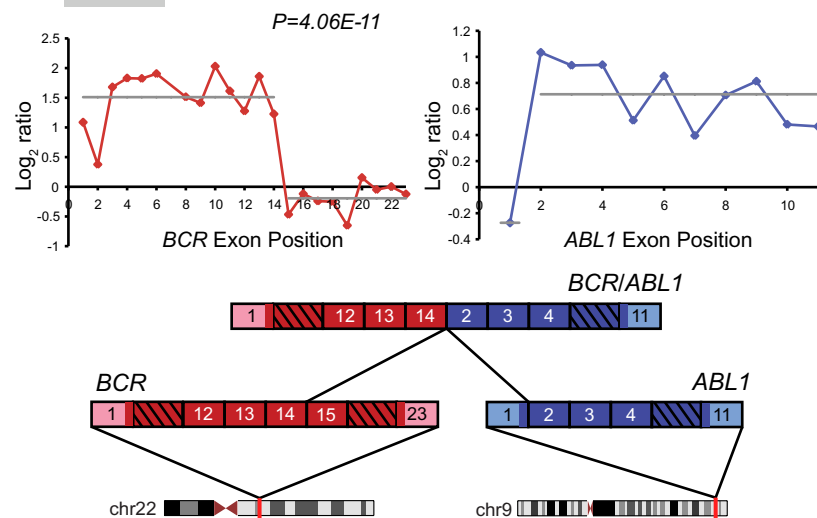

B

SUDHL-1

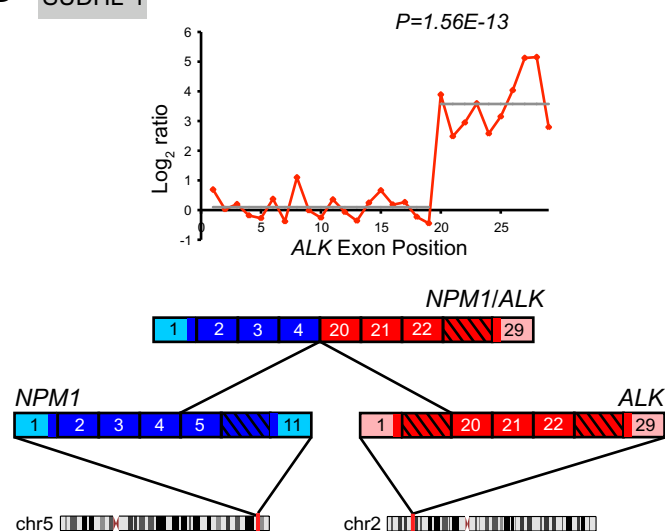

C

EOL-1

$P=2.18E-5$

$P=5.35E-6$

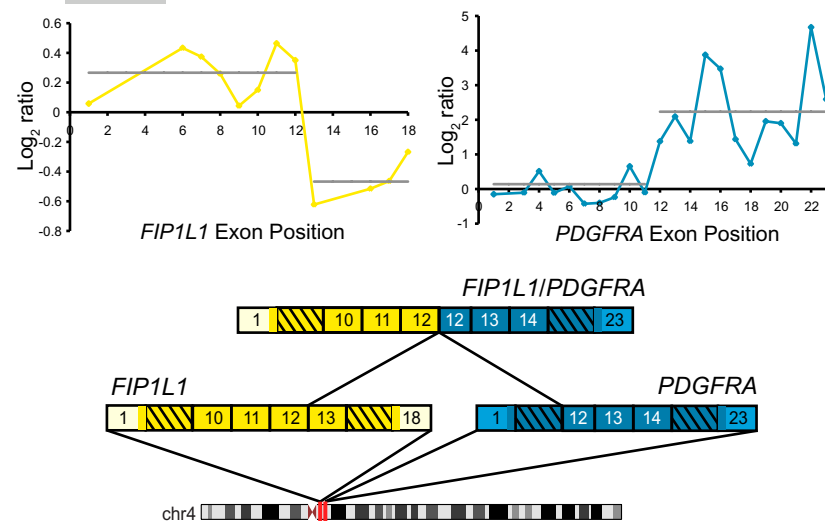

D

TPC-1

$P=1.82E-8$

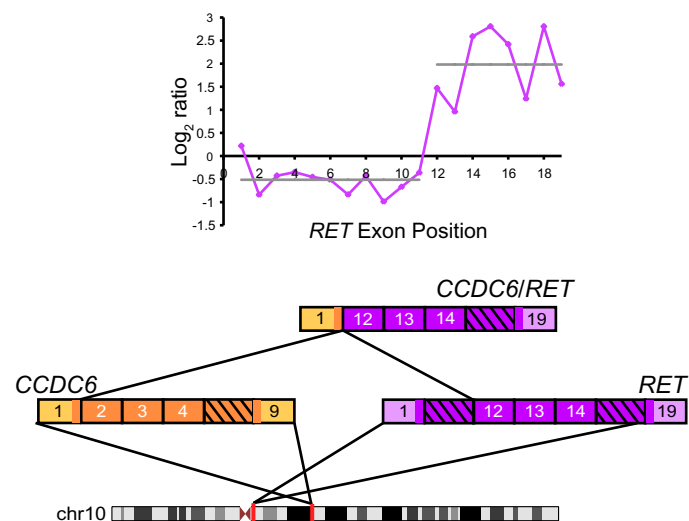

E

ALL-SL

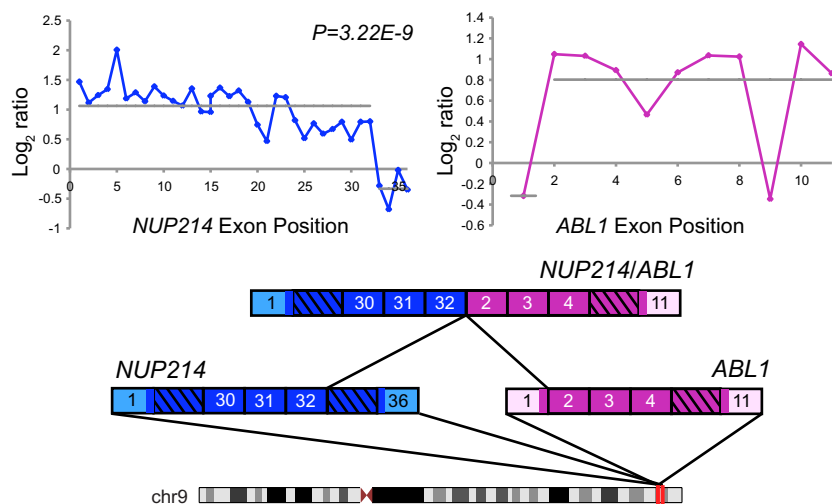

F

SKES-1

$P=3.45E-3$

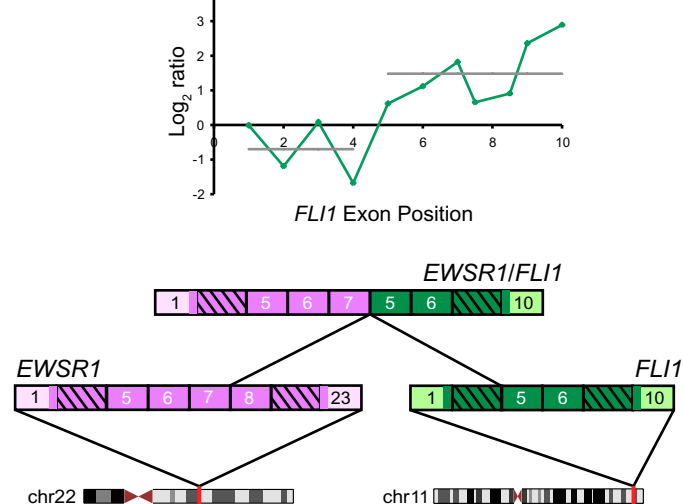

Supplement: Figure S4 — RBA rediscovery of known gene fusions in various cancers. Exonic expression breakpoints representing known gene fusions including (A) BCR/ABL1 in K562 (CML), (B) NPM1/ALK in SUDHL-1 (ALCL), (C) FIP1L1/PDGFRA in EOL-1 (eosinophilic leukemia), (D) CCDC6/RET in TPC-1 (thyroid cancer), (E) NUP214/ABL1 in ALL-SIL, (F) EWSR1/FLI1 in SKES-1 (Ewing sarcoma). (PDF) [file pgen.1003464.s004.pdf]

Figure S5

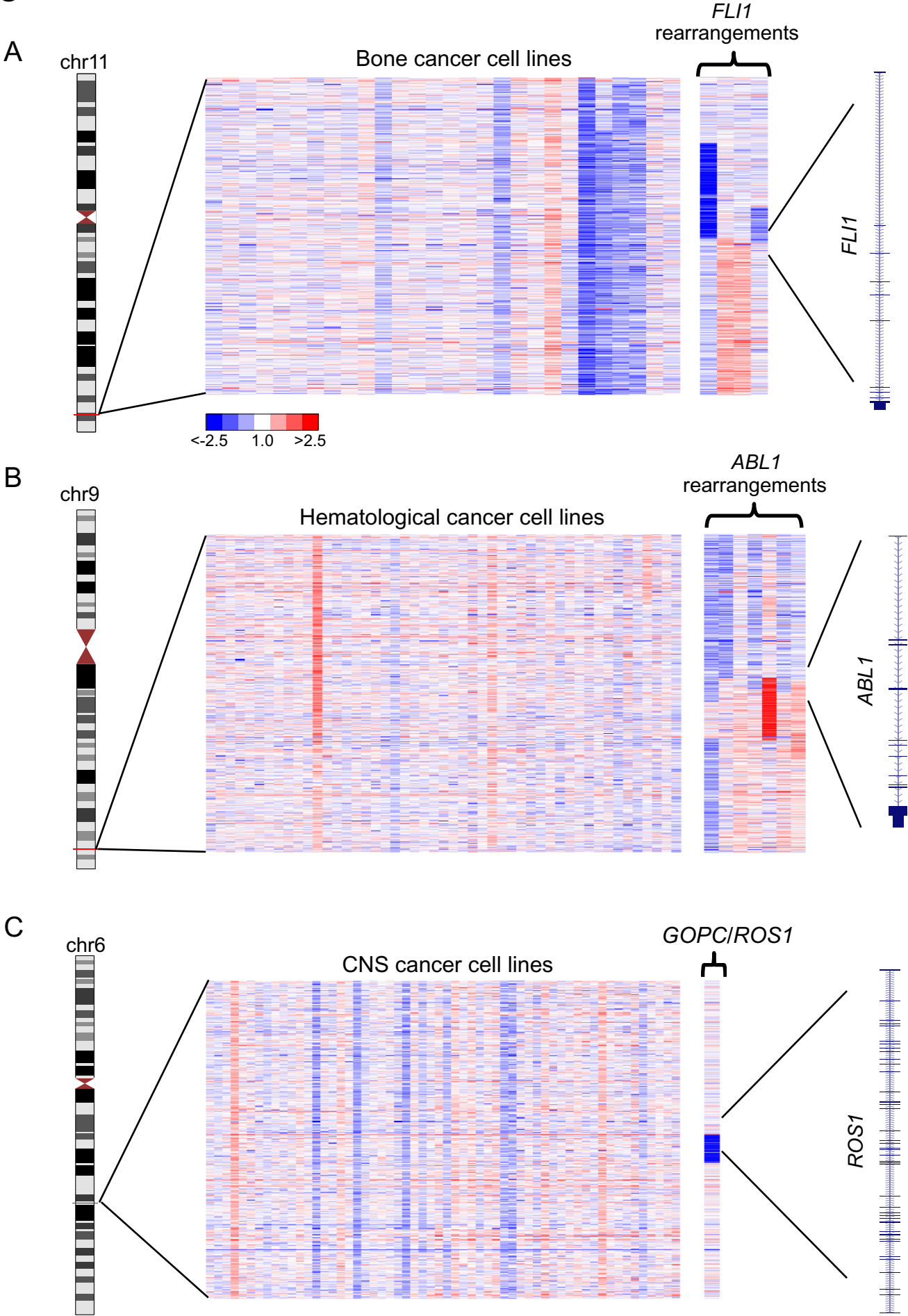

Supplement: Figure S5 — DBA rediscovery of known gene fusions in various cancers. (A) Heatmaps depicting identified intragenic breakpoints disrupting (A) FLI1 in four Ewing's sarcoma cell lines (EWSR1/FLI1), (B) ABL1 in seven CML (BCR/ABL1) and T-ALL (NUP214/ABL1) cell lines, and (C) ROS1 in glioblastoma cell line U-118MG (GOPC/ROS1). Samples without rearrangement are also depicted for comparison. (PDF) [file pgen.1003464.s005.pdf]
